# Cooperative effects of Janus and Aurora kinase inhibition by CEP701 in cells expressing Jak2V617F

**DOI:** 10.1111/jcmm.12005

**Published:** 2013-01-10

**Authors:** Karoline Gäbler, Catherine Rolvering, Jakub Kaczor, René Eulenfeld, Sergio Álvarez Méndez, Guy Berchem, Valérie Palissot, Iris Behrmann, Claude Haan

**Affiliations:** a Life Sciences Research Unit – Signal Transduction Laboratory, University of LuxembourgLuxembourg, Luxembourg; b MRC Clinical Sciences Centre, Faculty of Medicine – Cancer Genomics Group, Imperial CollegeLondon, UK; c Institute of Biology – Department of Systems Biology, Otto-von-Guericke-UniversityMagdeburg, Germany; d Department of Oncology – Laboratory of Experimental Hemato-Oncology, CRP SantéLuxembourg, Luxembourg

**Keywords:** Janus kinases, Jak2V617F, Aurora kinases, kinase inhibitors, MPN, CEP701

## Abstract

The Janus kinase 2 mutant V617F occurs with high frequency in myeloproliferative neoplasms. Further mutations affecting the Janus kinase family have been discovered mostly in leukaemias and in myeloproliferative neoplasms. Owing to their involvement in neoplasia, inflammatory diseases and in the immune response, Janus kinases are promising targets for kinase inhibitor therapy in these disease settings. Various quantitative assays including two newly developed screening assays were used to characterize the function of different small-molecule compounds in cells expressing Jak2V617F. A detailed comparative analysis of different Janus kinase inhibitors in our quantitative assays and the subsequent characterization of additional activities demonstrated for the first time that the most potent Jak2 inhibitor in our study, CEP701, also targets Aurora kinases. CEP701 shows a unique combination of both activities which is not found in other compounds also targeting Jak2. Furthermore, colony forming cell assays showed that Janus kinase 2 inhibitors preferentially suppressed the growth of erythroid colonies, whereas inhibitors of Aurora kinases preferentially blocked myeloid colony growth. CEP701 demonstrated a combined suppression of both colony types. Moreover, we show that combined application of a Janus and an Aurora kinase inhibitor recapitulated the effect observed for CEP701 but might allow for more flexibility in combining both activities in clinical settings, *e.g*. in the treatment of myeloproliferative neoplasms. The newly developed screening assays are high throughput compatible and allow an easy detection of new compounds with Janus kinase 2 inhibitory activity.

## Introduction

In 2005, a somatic activating point mutation in Janus kinase 2 (Jak2)—Jak2V617F was discovered in Philadelphia chromosome-negative myeloproliferative neoplasm (MPN) patients [1–4, 5]. Almost every patient suffering from polycythemia vera (PV) carries this mutation and it is found in about 50% of essential thrombocythemia (ET) and primary myelofibrosis (PMF) patients. These haematopoietic disorders are characterized by an overproduction of mature-appearing blood cells of one or more lineages and can lead to failure of the bone marrow and ineffective haematopoiesis [6]. Further genetic alterations affecting all members of the Janus kinase family (Jak1, Jak2, Jak3 and Tyk2) have been discovered in leukaemias and other cancers since 2005 [7]. Many of these mutations have been shown to constitutively activate the Jaks, resulting in cytokine hypersensitivity and constitutive activation of signal transducers and activators of transcription (STAT) 3 and 5, mitogen-activated protein (MAP) kinases and the phosphoinositide-3-kinase/protein kinase B (PI3K/AKT) pathway [2, 8–13]. As Janus kinases play a critical role in the pathogenesis of MPN and different other cancers (*e.g*. leukaemias, multiple myeloma) but also in inflammatory disorders (*e.g*. rheumatoid arthritis, psoriasis) and in the setting of allograft transplant rejection, the interest in developing small-molecule inhibitors of the Jak family has concomitantly risen. In the last few years new and potent inhibitors of Janus kinases (mostly ATP-competitive compounds) have been developed for different clinical applications (reviewed in [7, 14, 15]). A number of compounds have entered clinical trials [16, 17] and INCB018424, a Jak1 and Jak2 targeting compound, was recently approved by the FDA and the European Commission for treatment of myelofibrosis. However, the survival rate of myelofibrosis patients treated with INCB018424 is not different from patients treated with standard therapy [18, 19] and it is still ambiguous if the Jak2V617F allele burden is effectively improved upon Jak2 inhibitor treatment. Thus, the discovery of new compounds with Jak2 inhibitory activity as well as the development of alternative treatment strategies is highly required.

Here, we developed two new intact cell fluorescent screening procedures, which allow a reproducible monitoring of Jak2 activity, based on a comparative investigation of several described Jak(2) inhibitors in the cellular context. Some of the compounds did not inhibit Jak2V617F, and also the Jak-targeting inhibitors showed different potencies in intact cell assays. Furthermore, we show for the first time that the potent Jak inhibitor CEP701, which is tested in clinical trials, inhibits also Aurora kinases. CEP701 shows a unique combination of both activities, which is not found in other Aurora kinase inhibitors also targeting Janus kinases (*e.g*. AT9283). Moreover, we show that a combined application of Jak2 inhibitors with Aurora kinase inhibitors recapitulates the effects observed for CEP701 and might be beneficial for the treatment of MPN patients if CEP701 fails clinical approval.

## Materials and methods

### Materials

All compounds were dissolved in DMSO at a concentration of 10 mM. For the experiments the stock solutions of the inhibitors were diluted in the respective cell culture media. From Symansis (Auckland, New Zealand) we purchased JNJ7706621, TG101209, TG101348 and VX680. From Synkinase (San Diego, CA, USA) we acquired CYT387 and VX680. Calbiochem (Merck4Biosciences, Darmstadt, Germany) provided AG490, JAK Inhibitor 1 (JI1, pyridone 6, CMP6) and WP1066. Selleck Chemicals (Houston, TX, USA) provided AT9283 and VX680. CEP701 was from Tocris Bioscience (Bristol, UK). LFM-A13 was purchased from Axxora (San Diego, CA, USA), Sunitinib was from Cayman Chemical Company (Ann Arbor, MI, USA) and TG101348 was also provided by Axon Medchem (Groningen, The Netherlands).

### Cell culture

The HEL (human erythroleukaemia) cell line was purchased at Leibniz Institute DSMZ (Braunschweig, Germany). The cells were amplified, aliquots were frozen and after thawing the cells were cultivated for 2 months at most. The cells were maintained in RPMI–1640 medium (Lonza, Basel, Switzerland) supplemented with 10% FBS, 100 mg/l streptomycin, 60 mg/l penicillin and 2 mM L-glutamine. For details on the cultivation of the other cell lines see the Supporting Information.

### Cell lysis and Western blot analysis

All steps of cell lysis were performed on ice. HEL cells were either lysed with Mammalian Protein Extraction Reagent (M-PER, Thermo Fisher Scientific, Waltham, MA, USA), which was supplemented with 4× Laemmli buffer or directly in 1× Laemmli buffer. After denaturation, the samples were run on SDS polyacrylamide gels, transferred to a PVDF (Carl Roth, Karlsruhe, Germany) or nitrocellulose membrane (GE Healthcare, Little Chalfont, UK) and probed with the respective antibodies. The anti-phospho-histone H3, anti-histone H3, anti-phospho-Jak2, anti-Jak2 and anti-phospho-STAT3 antibodies were purchased from Cell Signalling Technology (Danvers, MA, USA). The anti-phospho-Jak1, anti-Jak3, anti-STAT5 and anti-tubulin antibodies were obtained from Santa Cruz Biotechnology (Santa Cruz, CA, USA). The anti-phospho-STAT5 and anti-STAT3 antibodies were purchased from BD Biosciences (Franklin Lakes, NJ, USA), whereas the anti-GFP antibody (used to detect STAT3-YFP) was from Rockland Immunochemicals (Gilbertsville, PA, USA). The anti-phospho-Jak1 antibody was used to detect phosphorylated Jak3 as described previously [20]. This is possible because the antibody is cross-reactive and Jak1 (130 kD) and Jak3 (110 kD) are well separated by SDS polyacrylamide gel electrophoresis. The horseradish peroxidase-conjugated secondary antibodies were purchased from Cell Signalling Technology. Luminescent signals were detected using an ECL solution as described before [21]. Secondary antibodies IRDye® 700 and 800, used for the fluorescent Western blot detection with the Odyssey® Infrared Imaging System (LI-COR Biosciences, Lincoln, NE, USA), were obtained from LI-COR Biosciences. Quantitation was performed with the analysis software V3.0 provided by LI-COR Biosciences. IC_50_ values were determined using GraphPad Prism 5.01, log [inhibitor] *versus* response-Variable slope 4PL curve fit from biological replicates (*n* = 4–12).

### Cell proliferation assay

Human erythroleukaemia cells were cultured with inhibitor at indicated concentrations or left untreated for 72 hrs. Cells were washed and resuspended in FACS buffer (PBS, 5% FBS, 0.1% NaN_3_) containing 15,000 phycoerythrin-labelled Calibrite™ Beads per ml (BD Biosciences) and 1 μM SYTOX® Blue Dead Cell Stain (Invitrogen, Life Technologies, Carlsbad, CA, USA) and incubated for 5 min. on ice. Samples were run on a FACSCanto II flow cytometer (BD Biosciences) and analysed using FACSDiva (BD Biosciences) software. The amount of inhibitor-treated cells was calculated as percentage of maximum number of cells (= untreated control). IC_50_ values were determined using GraphPad Prism 5.01, log [inhibitor] *versus* response-Variable slope 4PL curve fit from biological replicates (n = 3–5).

### pSTAT-ZsGreen reporter gene assay

HEK-FRT-TO-HAEpoR-Jak2V617F cells stably integrating the pSTAT-ZsGreen plasmid, henceforth called “HEK-V617F-STAT-Rep.” cells, were treated with 10 ng/ml doxycycline to induce expression of Jak2V617F for 24 hrs and were additionally treated with 7500, 2500, 833, 277.8, 92.6, 30.8, 10.3, 3.4 or 1.1 nM of the different inhibitors. The cells were harvested using trypsin-EDTA, washed and resuspended in FACS buffer and analysed on a FACSCanto II flow cytometer. The fluorescence signal of the sample containing 1.1 nM of inhibitor was set to 100%. IC_50_ values were determined using GraphPad Prism 5.01, log [inhibitor] *versus* response-Variable slope 4PL curve fit from biological replicate experiments (n = 4).

### STAT3-YFP translocation assay

γ2A-FRT-TI-Jak2V617F/STAT3-YFP cells were seeded on 96-well glass bottom plates (Matrical Bioscience, Spokane, WA, USA) and induced with 5 μg/ml doxycycline. Different concentrations of inhibitors (2, 6, 18, 54, 162, 486, 1458 nM) were added after 24 hrs of doxycycline treatment for another 12–24 hrs. After staining the cells with the DNA dye Hoechst 33342 (Invitrogen, Life Technologies) at a concentration of 1 μg/ml for 20 min., the cells were washed with PBS and fixed using 4% paraformaldehyde (PFA). Finally, PBS was added to each well and automated confocal cell imaging of the cells on 96-well plates was performed using a LSM 510 inverted laser scanning microscope (Carl Zeiss AG, Oberkochen, Germany). YFP was detected with λ_exc_ = 514 nm and λ_em_ = 530–600 nm, the Hoechst 33342-stained nuclei were recorded with λ_exc_ = 405 nm and λ_em_ = 420–490 nm. Quantitation of YFP signals was performed using the cell image analysis software “Cell Profiler” (http://www.cellprofiler.org) [22, 23]. Briefly, the nuclei shape was determined automatically and the amount of YFP fluorescence that colocalized with the Hoechst 33342-stained nuclei was determined. The nuclear YFP signal intensities were normalized with respect to overall YFP signal intensities to account for differences in STAT3-YFP expression that can occur. IC_50_ values were determined using GraphPad Prism 5.01, log [inhibitor] *versus* response-Variable slope 4PL curve fit from biological replicate experiments (n = 3–8) performed each time in technical quadruplicates.

### Cell cycle analysis

Human erythroleukaemia cells were cultivated with inhibitor at indicated concentrations or left untreated for 24 and 48 hrs. Cells were washed once with PBS and fixed in 70% ethanol over night at 4°C. Fixed cells were washed with PBS and then resuspended in PBS containing 50 μg/ml propidium iodide and 50 μg/ml RNase and incubated at 37°C for 15 min. Cell cycle profiles were recorded on a FACSCanto II flow cytometer.

### Colony forming cell assay

All experiments on patient samples were approved by the Comité National d'Etique de Recherche (CNER) in Luxembourg according to the Declaration of Helsinki. An informed, written consent of every patient included in the study has been obtained.

Peripheral blood mononuclear cells (PBMC) from Jak2V617F-positive MPN patients were isolated by a Ficoll-Paque PLUS (GE Healthcare) gradient centrifugation according to the manufacturer's instructions. CD34^+^ cells were purified using the CD34 MicroBead Kit on LS columns and a QuadroMACS Separator (all from Miltenyi Biotec, Bergisch Gladbach, Germany) according to the manufacturer's protocol. The CD34^+^ cells (500 cells per 35 mm dish) were seeded in methylcellulose medium MethoCult® H4230 (StemCell Technologies, Grenoble, France) supplemented with 1 U/ml Epo (Calbiochem, Merck4Biosciences), 10 ng/ml IL-3, 10 ng/ml G-CSF and 10 ng/ml GM-CSF (all from PeproTech, Rocky Hill, NJ, USA) according to the manufacturer's instructions. Cells were incubated with inhibitors at indicated concentrations or left untreated. The different colony types (CFU-E, BFU-E and CFU-GM) were counted using an inverted microscope after 12–16 days of culture. The amount of colonies grown from inhibitor-treated cells was calculated as percentage of maximum number of colonies (= untreated control). Replicates with cells isolated from three patients were performed.

## Results

### Some of the proposed Jak inhibitors do not target Jak2 but suppress cell growth

We chose 12 compounds (AT9283, CEP701, TG101209, TG101348, CYT387, JI1, VX680, Sunitinib, WP1066, AG490, LFM-A13 and JNJ7706621, see Figure S1) with proposed Jak2 inhibitory activity and/or reported growth inhibitory activity towards cells from MPN patients for comparative testing using different read-outs and cell systems.

**Fig. 1:**
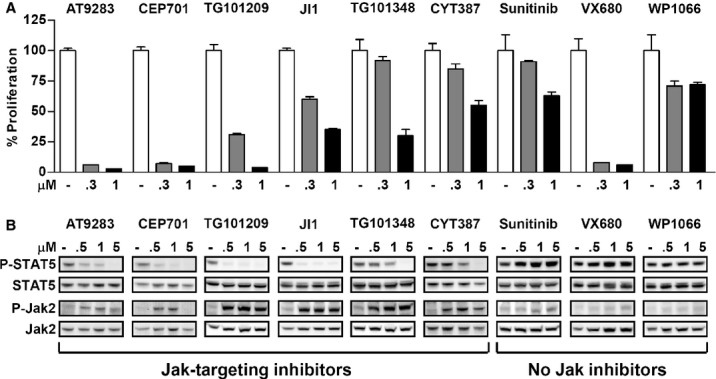
Effect of the different inhibitors on proliferation of HEL cells and Jak2V617F signal transduction. (**A**) HEL cells were treated with different compounds at concentrations of 0.3 or 1 μM or left untreated for 72 hrs. One representative biological replicate is shown. (**B**) HEL cells were treated with 0.5, 1 or 5 μM of the different inhibitors or left untreated for 3 hrs. The expression and phosphorylation state of STAT5 (P-STAT5) and Jak2 (P-Jak2) was assessed by Western blot immunodetection.

Human erythroleukaemia cells endogenously expressing the constitutively active mutant Jak2V617F were used to assess the effect of the compounds on proliferation (Fig. 1A). Furthermore, the actual capacity of the different compounds to inhibit Jaks was evaluated by monitoring phosphorylation of STAT5, a direct target of Jak2V617F which is constitutively phosphorylated in HEL cells (Fig. 1B and Figure S2C). Several compounds such as AT9283, CEP701, TG101209 and JI1 reduced proliferation of HEL cells very effectively and at the same time strongly reduced phosphorylation of STAT5 at a concentration of 0.5 μM. TG101348 and CYT387 showed the same effects but were less efficient. Six of the 12 compounds (VX680, Sunitinib, WP1066, AG490, LFM-A13 and JNJ7706621) did not inhibit the phosphorylation of STAT5 up to concentrations of 5 μM (Fig. 1B and Figure S2C). AG490, LFM-A13 and JNJ7706621 neither affected cell growth at concentrations up to 1 μM (data not shown). However, Sunitinib and WP1066 had a moderate effect on HEL cell growth, while VX680 very efficiently inhibited HEL cell growth already at 0.3 μM.

The effects of these compounds were validated in other cellular systems including the UKE-1 (endogenously expressing Jak2V617F [24]) and BA/F3-EpoR-Jak2V617F [10] cell lines (Figure S2A and B). The phosphorylation of STAT5 was efficiently decreased upon treatment with JI1, TG101209, TG101348, AT98283, CEP701 and CYT387, whereas Sunitinib, VX680, WP1066 and AG490 did not inhibit STAT5 phosphorylation. Furthermore, two potent Jak2 inhibitors (TG101209, CEP701) and VX680 inhibited very efficiently the cell proliferation of UKE-1 and BA/F3-EpoR-Jak2V617F cells.

### The Jak2 phosphorylation status is increased upon Jak inhibition in HEL cells

Treatment with compounds targeting Jak2V617F (AT9283, CEP701, TG101209, TG101348, CYT387 and JI1) led to an increased phosphorylation of Jak2 (Fig. 1B), although the Jak2V617F-mediated phosphorylation of STAT5 was prevented by the inhibitor treatment. The intensity of the hyper-phosphorylation and the inhibitor concentration, at which it occurs, are reproducible for each inhibitor. Moreover, the effect of hyper-phosphorylation upon Jak inhibitor treatment was not restricted to Jak2V617F in HEL cells. In HEK-EpoR cells doxycycline-inducibly expressing the Jak2V617F or Jak2K539L [8] mutant an increase in Jak-mutant phosphorylation was observed upon treatment with JI1 (Figure S2D). Hyper-phosphorylation of Jak2V617F in HEL cells was also observed after treatment with CP690,550 (another pan-Jak inhibitor, Figure S2E). Moreover, in CMK cells expressing the constitutively active Jak3 mutant A572V (a cell line which was not further characterized in this study) hyper-phosphorylation of Jak3A572V was also observed after CP690,550 treatment (Figure S2F). Interestingly, the increase in Jak2 and Jak3 phosphorylation in the aforementioned cellular systems was not observed for the compounds, which did not inhibit the phosphorylation of STAT5 (VX680, Sunitinib, WP1066, AG490, LFM-A13 and JNJ7706621; Fig. 1B and Figure S2C and F). Taken together, the Jak2 phosphorylation state upon inhibitor treatment does not necessarily reflect the Jak2 activation status and is thus not a suitable parameter to monitor the efficacy of a Jak-targeting ATP-competitive compound.

### Jak2V617F activity or inhibition can be effectively monitored by two new assays using fluorescent read-outs of STAT activation

Investigating the efficacy of inhibitors using Western blot detection is not applicable for high throughput analysis. Therefore, we developed intact cell assays, which are easy to handle and high throughput compatible to monitor Jak2 activity: we here describe the first Jak2V617F-controlled STAT-reporter gene assay using the fluorescent read-out protein ZsGreen. In addition, we measured STAT3-YFP translocation to monitor Jak2V617F activity in a confocal microscopy assay. As both assays utilize fluorescent proteins, complex treatments are not required prior to the measurements.

**Fig. 2:**
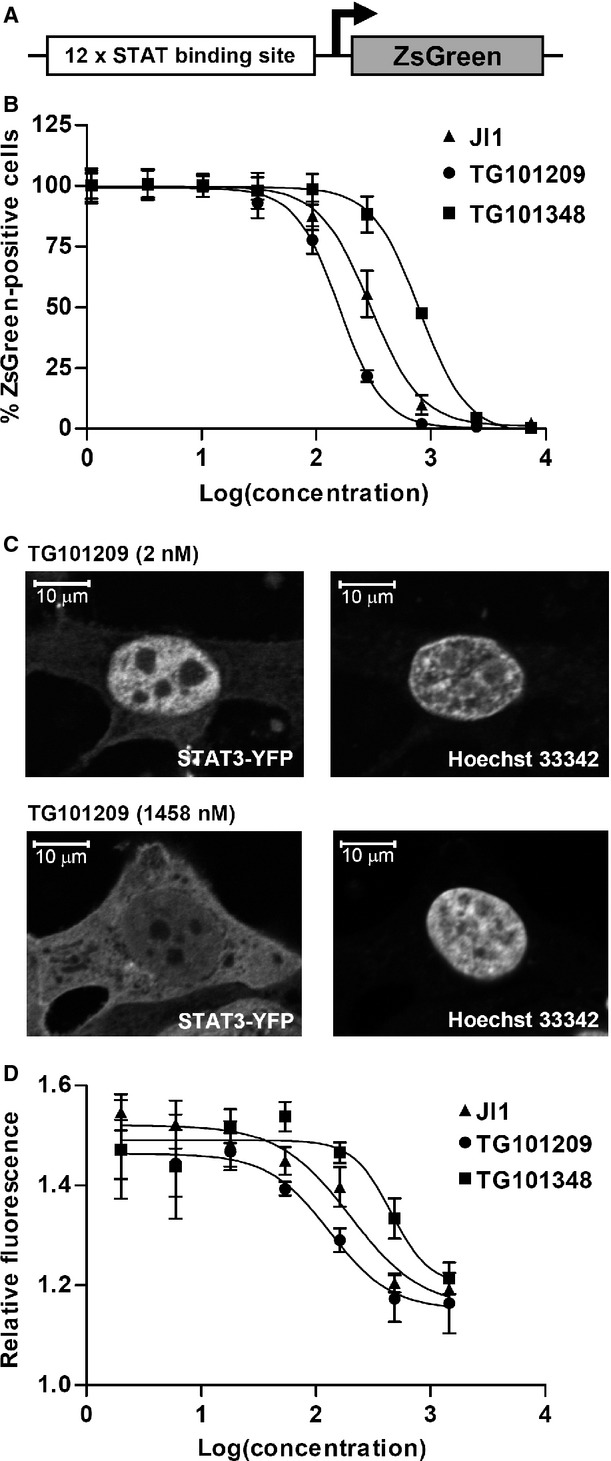
Two newly developed fluorescent intact cell screening assays allow detecting differences in efficiency between the various Jak inhibitors. (**A**) Schematic representation of the STAT-responsive reporter gene construct. (**B**) HEK-V617F-STAT-Rep. cells were treated with 7500, 2500, 833, 278, 93, 31, 10.3, 3.4 or 1.1 nM of the different inhibitors for 24 hrs. The fluorescent reporter gene product was measured by flow cytometry. Overlays of the curves derived from three independent biological triplicates for the indicated inhibitors are shown. (**C**) γ2A-FRT-TI-Jak2V617F/STAT3-YFP cells were treated with 2 or 1458 nM of TG101209 for 12 hrs. Upper panels: The nuclear localization of phosphorylated STAT3-YFP and Hoechst 33342-stained nuclei are shown for a concentration of 2 nM of TG101209, when Jak2V617F is not inhibited. Lower panels: The predominant cytoplasmic localization of non-phosphorylated STAT3-YFP is shown for a concentration of 1458 nM of TG101209, when Jak2V617F is inhibited. The nuclear staining by Hoechst 33342 remains unchanged. (**D**) γ2A-FRT-TI-Jak2V617F/STAT3-YFP cells were treated with 2, 6, 18, 54, 162, 486 or 1458 nM of the different inhibitors for 12 hrs.

The HEK-V617F-STAT-Rep. cells harbour a stably integrated ZsGreen reporter gene construct which contains 12 STAT consensus sequences in its promoter region (see Fig. 2A and Supporting Information). Jak2V617F is induced upon doxycycline treatment of the cells leading to phosphorylation of STAT5 and STAT3 (Figure S3A) [8], which in turn results in the expression of the fluorescent ZsGreen protein (Figure S3B and C). Jak inhibitor treatment prevented STAT phosphorylation (Figure S3A) which in turn resulted in decreased expression of the fluorescent protein, which was monitored by flow cytometry (Fig. 2B and Figure S3D) or alternatively using a fluorimeter (data not shown).

The γ2A-FRT-TI-Jak2V617F/STAT3-YFP cells, doxycycline-inducibly expressing STAT3-YFP and Jak2V617F (Figure S4A and B), were used in the confocal imaging assay. Jak2V617F phosphorylates STAT3-YFP which then translocates into the nucleus (Fig. 2C). Jak inhibitor treatment led to decreased levels of STAT3-YFP phosphorylation (Figure S4C) which in turn resulted in decreased translocation of STAT3-YFP into the nucleus (Fig. 2C). The phosphorylation-dependent STAT3-YFP nuclear translocation was quantitated upon inhibitor treatment using the ‘Cell Profiler’ software (see Figure S4D). Nuclear localization of STAT3-YFP was suppressed, when Jak2V617F was inhibited by JI1, TG101209, TG101348, AT9283, CEP701 and CYT387 (Fig. 2C and D; Figure S4D and E and Table 1) but was not observed for compounds not targeting Jak2V617F.

**Table 1:** Effects of the different compounds used in this study

	IC_50_ values (nM)*				
	
Compound	*In vitro* kinase assay†	WB (P-STAT5)‡	STAT-reporter gene assay	STAT3-YFP translocation assay	Proliferation assay
JI1	1.6	133 ± 62	291 ± 40	197 ± 73	506 ± 18
TG101209	0.76	92 ± 18	156 ± 10	125 ± 45	243 ± 41
TG101348	11	636 ± 203	850 ± 101	385 ± 61	725 ± 85
CYT387	1.5	633 ± 90	794 ± 7	459 ± 126	1180 ± 413
AT9283	0.88	309 ± 126	105 ± 15	74 ± 38	29 ± 5
CEP701	1.2	97 ± 39	111 ± 22	45 ± 15	81 ± 20
VX680	480	No inhibition	No inhibition	No inhibition	158 ± 26
WP1066	–	No inhibition	No inhibition	–	n.d.§
Sunitinib	2400	No inhibition	–	–	n.d.§
JNJ7706621	–	No inhibition	–	–	n.d.§
AG490	–	No inhibition	–	–	–
LFM-A13	–	No inhibition	–	–	–

* The IC_50_ values (mean ± SD) determined from biological replicates in the different assays are shown.

† The Jak2 *in vitro* kinase assays were performed by Caliper Discovery Alliances & Services. Exact assay conditions are described in the Supporting Information.

‡ Phospho-STAT5 (P-STAT5) levels in HEL cells were assessed by Western blot immunodetection.

§ IC_50_ values were not determined as these compounds did not show sufficient growth reduction in the tested concentration range.

### *In vitro* kinase assay data lack accuracy in predicting inhibitor behaviour in intact cells

Comparison of the *in vitro* kinase assay data of JI1, TG101209, TG101348, CEP701, CYT387 and AT9283 with the IC_50_ values derived from Western blot analysis of phospho-STAT5 signals in HEL cells revealed discrepancies in the behaviour of the compounds in these two assays (Table 1, Figure S5A). As expected, higher concentrations of the compounds were needed in the cellular assays to compete with the intracellular ATP concentrations (1–5 mM). Besides this, the five compounds showing IC_50_ values between 0.76 and 1.5 nM (JI1, TG101209, CYT387, AT9283 and CEP701) in the *in vitro* kinase assay showed a considerably larger range of IC_50_ values in the cellular assays. CYT387 had significantly higher IC_50_ values in all intact cell assays compared with JI1, both being equipotent in the *in vitro* kinase assay. On the other hand, TG101348 and CYT387 have roughly the same activity in intact cell assays, while CYT387 was approximately one order of magnitude more potent in the *in vitro* kinase assay. As a conclusion, the exact activity of a compound in cells is hard to predict from its activity in the *in vitro* kinase assay, showing the need to combine both technologies to clearly uncover the activity of a certain compound on its respective target kinases. A comparison of our *in vitro* kinase data with those previously published is shown in Figure S5B.

### A detailed comparative study of a selection of compounds

We further investigated a selection of compounds and compared the IC_50_ values determined from two reference assays (Western blot analysis of phospho-STAT5 and proliferation assays in Jak2V617F-dependently growing HEL cells) with the IC_50_ values obtained in the new fluorescent assays (Table 1). The efficacy of JI1, TG101209, TG101348 and CYT387 within the assays was always comparable. In general, the concentrations to achieve half-maximal inhibition are slightly higher in the fluorescent reporter gene assay than those in the confocal imaging assay or Western blot analysis. This variation between assays may reflect differences in Jak2V617F expression in the different cell systems. For the Jak inhibitors JI1, TG101209, TG101348 and CYT387, we show that the IC_50_ values for growth inhibition are generally higher than those determined from Western blot experiments tracing STAT5 phosphorylation (Table 1). As seen in Figure 1 and in Table 1 again, discrepancies between IC_50_ values determined by phospho-STAT5 measurements (Western blot analysis) or growth inhibition can reveal inhibitors, which have a different mode of action than the inhibition of Jak2 (*e.g*. VX680). Interestingly, also the Jak inhibitors AT9283 and CEP701 showed differences when comparing the IC_50_ values of Western blot analysis, our new assays and proliferation assays (Table 1). For these two compounds the IC_50_ values in our novel assays and the growth assay were lower than expected from the Western blot experiments. This at first glance surprising behaviour sparked a more detailed investigation of these effects as described below.

### CEP701 has Aurora kinase-inhibitory potential

**Fig. 3:**
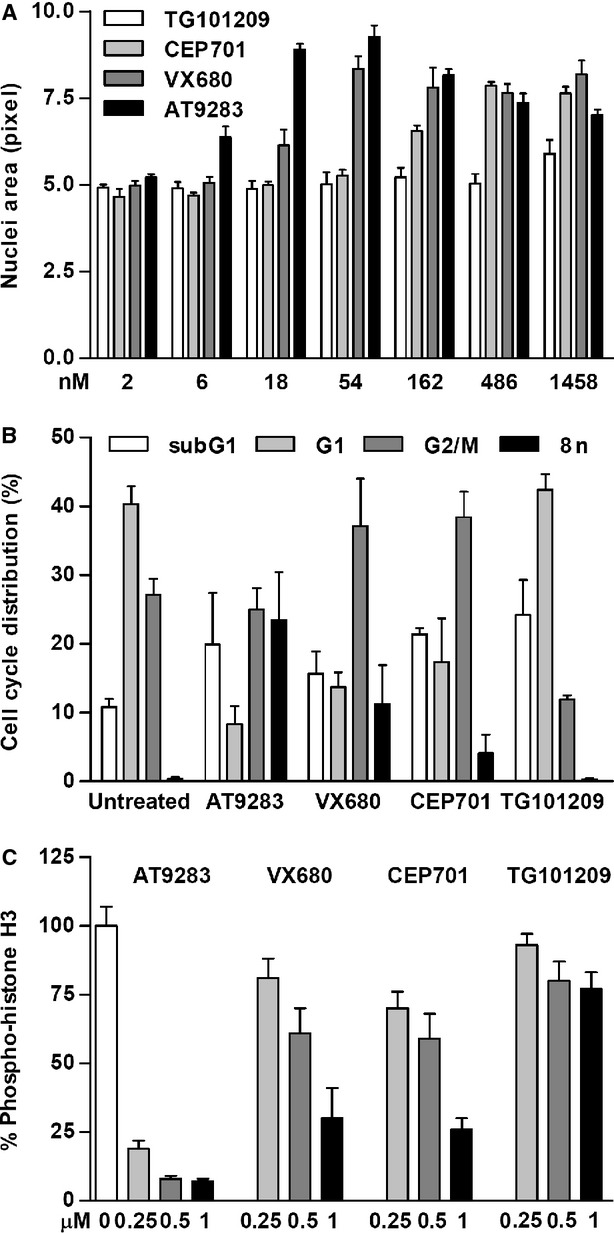
CEP701 has Aurora kinase-inhibitory activity (**A**) γ2A-FRT-TI-Jak2V617F/STAT3-YFP cells were treated with the indicated inhibitors for 24 hrs. The cells were analysed by confocal imaging. The increase in nuclear size as determined with the software ‘Cell Profiler’ is shown for CEP701, AT9283 and VX680 from one representative biological replicate. TG101209 is shown as negative control. (**B**) HEL cells were treated with 500 nM CEP701, VX680, TG101209 or 100 nM AT9283 for 24 hrs and then subjected to cell cycle analysis. The relative proportion (in %) of cells constituting the subG1 = apoptotic (white bars), G1 = 2n (light grey bars), G2/M = 4n (dark grey bars) or 8n (black bars) peaks are shown. The mean ± SD from three biological replicates is shown. (**C**) HEL cells were treated with 100 ng/ml of Nocodazole for 16 hrs to block the cells in the M phase of cell cycle and afterwards incubated with different concentrations (0.25, 0.5 or 1 μM) of CEP701, VX680, AT9283 and TG101209 or left untreated for 3 hrs. Relative phospho-histone H3 levels were assessed by quantitative Western blot immunodetection.

Based on the confocal images of the STAT3-YFP translocation assay the software ‘Cell Profiler’ allows the analysis of many parameters in parallel. Taking advantage of this we found that treatment with AT9283, VX680 and CEP701 led to an increase in nuclei size (Fig. 3A and Figure S6A) at 6, 18 and 162 nM respectively, while TG101209 did not show this effect. When treating cell lines not expressing Jak2V617F (2C4, HCT-116 and K-562 cells) with VX680 and CEP701, this effect was also clearly visible (Figure S6B). This indicates that the increase in nuclear size is independent of Jak2V617F.

As AT9283 and VX680 are known to inhibit Aurora kinases [25, 26], we analysed these two compounds and CEP701 using cell cycle analysis. A typical effect of Aurora kinase inhibition is the emergence of an 8n peak in addition to the 2n (G1 phase) and 4n (G2/M phase) peaks normally observed in growing cell cultures [27]. Jak inhibitors (TG101209 in Fig. 3B and Figure S6C) are known to inhibit growth of HEL cells by inducing G1 arrest [28], reflected by the reduction in the G2/M peak intensity (dark grey bars in Fig. 3B). AT9283, VX680 and CEP701, however, all showed the emergence of an 8n peak (black bars in Fig. 3B) and an increase in the 4n/2n ratio (compare light and dark grey bars in Fig. 3B, Figure S6C). AT9283 was the most efficient compound followed by VX680 and CEP701 in all related assays. HEL cells also increased in size with rising time of treatment owing to Aurora kinase inhibition by AT9283, VX680 and CEP701, whereas the effect was not observed for TG101209 (Figure S6D). Moreover, we investigated the effect of the compounds on phosphorylation of histone H3, a direct target of Aurora kinase B [29]. Reduction in histone H3 phosphorylation could be demonstrated for all three inhibitors, AT9283 again being the most efficient one followed by VX680 and CEP701. TG101209 had only very little effect (Fig. 3C). The decline of histone H3 phosphorylation upon treatment with VX680 or CEP701 could also be detected in UKE-1 and BA/F3-EpoR-Jak2V617F cells (Figure S6E). *In vitro* kinase assays showed that CEP701 indeed inhibited Aurora kinase A and B with similar IC_50_ values as AT9283 and VX680 (Figure S6F).

### Jak inhibitors preferentially suppress growth of erythroid colonies, whereas Aurora kinase inhibitors preferentially block myeloid colony growth

**Fig. 4:**
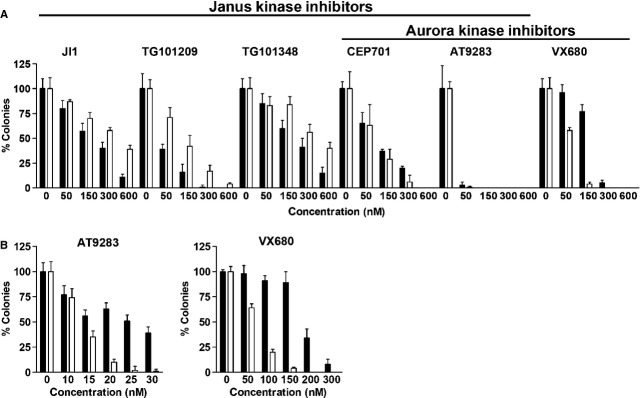
Aurora kinase and Jak2 inhibition has differential effects on cells of the erythroid and myeloid lineage in CFC assays. CD34^+^ cells of Jak2V617F-positive MPN patients were subjected to CFC assays in the absence or presence of kinase inhibitors at different concentrations. The amount of erythroid colonies (black bars) or myeloid colonies (white bars) grown from inhibitor-treated cells was calculated as percentage of maximum number of colonies in the untreated control. The results of one experiment are shown. The replicates were performed with cells isolated from three different patients.

We analysed the effect of several compounds in colony forming cell (CFC) assays. For this, the development of erythroid and myeloid colonies originating from haematopoietic progenitor cells (CD34^+^) derived from Jak2V617F-positive MPN patients was monitored. Jak inhibitors without additional Aurora kinase activity reduced erythroid and myeloid colony growth dose dependently, generally being more efficient in affecting erythroid colony numbers (Fig. 4A). Interestingly, AT9283 (Aurora kinase and Jak inhibitor) and VX680 (Aurora kinase inhibitor) reduced the numbers of myeloid colonies very efficiently but had less effect on erythroid colonies (Fig. 4). The same behaviour was also observed for other Aurora kinase inhibitors (AZD1152, Danusertib and SNS314, Figure S7). AT9283 was the most potent Aurora kinase inhibitor in our test, but VX680 was also very efficient: hardly any colony could be detected at concentrations of 50 or 300 nM. Among the Jak inhibitors with Aurora kinase-inhibitory potential, CEP701 showed an intermediate behaviour compared with the Jak or Aurora kinase inhibitors. Interestingly, the erythroid and myeloid colonies were equally well suppressed at a given concentration. The differential effects of Jak and Aurora kinase inhibitors on growth of myeloid colonies were also observed when CD34^+^ cells of healthy donors were subjected to CFC assays (data not shown).

### Jak and Aurora kinase inhibitors cooperate to suppress growth of Jak2V617F-expressing cells

**Fig. 5:**
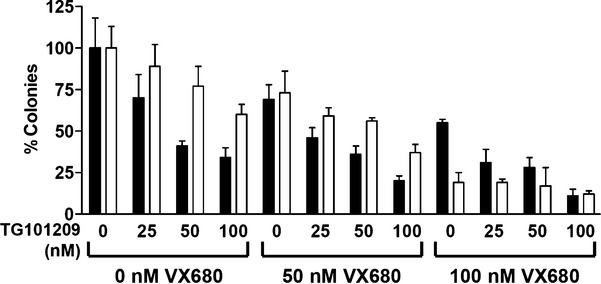
Jak and Aurora kinase inhibitors cooperate to suppress growth of Jak2V617F-expressing cells. CD34^+^ cells of Jak2V617F-positive MPN patients were subjected to CFC assays using the Aurora kinase inhibitor VX680 and the Jak2 inhibitor TG101209 in combination. The results of one experiment are shown. The replicates were performed with cells isolated from three different patients.

As Jak inhibitors rather suppressed growth of erythroid colonies, whereas Aurora kinase inhibitors preferentially blocked myeloid colony growth, we applied combinations of Jak and Aurora kinase inhibitors in the CFC assay. We observed that, also when applied in combination, the two types of inhibitors conserved their function: myeloid colony growth is preferentially suppressed dose dependently by VX680 and erythroid colony growth is preferentially suppressed dose dependently by TG101209 (Fig. 5) or TG101348 (Figure S8).

Furthermore, a drug combination analysis of VX680 and TG101209 according to the Chou-Talalay method (see Supporting Information) in BA/F3-EpoR-Jak2V617F cells was performed (Figure S9). The CI values range from 0.654 to 0.835 for a total dose of inhibitors of 0.55 to 1.27 μM indicating slight to moderate synergistic effects for this combination treatment. Only for the lowest concentration of compounds when the inhibitory effect on cell proliferation is less pronounced a CI value of 0.996 indicates an additive effect for the drug combination. We also find synergism when treating UKE-1 cells with VX680 and TG101209 in combination (data not shown).

## Discussion

In this comparative study of several proposed Janus kinase inhibitors, our aim was to characterize the compounds in depth in the cellular context, mainly concerning their activity towards Jak2. We also aimed at identifying reproducibly measurable parameters, which could be used to establish robust intact cell assays to monitor Jak2 activity.

Phosphorylation of Jak2 was not suitable to monitor the activation state of Jak2V617F upon inhibitor treatment as we observed that hyper-phosphorylation of Jak2 occurs upon treatment with any inhibitor targeting Jak2, although the inhibitors efficiently prevented STAT5 phosphorylation (Fig. 1B and Figure S2E; [8, 30]). The effect was also observed with Jak2K539L and Jak3A572V (Figure S2D and F) and has been described in analogue-sensitive mutants of Jak1 and Jak3 [20]. This ‘phosphorylation priming’ [31, 32] might result from a stabilization of the active conformation of the two Jaks in the cytokine/cytokine-receptor/Jak signalling complex. The Jaks might be able to trans-phosphorylate each other when the inhibitor is, at times, displaced competitively by ATP. However, the fast inhibitor displacement of ATP does not allow efficient signal transduction so that negative feedback mechanisms like recruitment of phosphatases or SOCS protein expression [8] are prevented. JI1 and CP690,550 are known to bind the kinase domains of Jak1, Jak2, Jak3 and Tyk2 in the active conformation (type I kinase inhibitors) [33–35]. The observed hyper-phosphorylation of Jak2V617F upon inhibitor treatment also suggests that in addition to JI1 and CP690,550, TG101209, TG101348, AT9283, CEP701 and CYT387 also bind the Jak2 kinase domain in the active conformation. It has been shown very recently that type II inhibitors binding the Jak2 kinase domain in the inactive conformation do not induce an increased Jak2 phosphorylation [36]. Considering the different Jak phosphorylation behaviour upon binding of a type I or II kinase inhibitor and the recently reported fact that Jaks do not necessarily need to be phosphorylated to be active and phosphorylate their substrates [20, 37], we conclude that Jak phosphorylation is not the ideal parameter to investigate Jak activity.

Jak2V617F has been described to activate multiple STAT factors (STAT5, STAT3 and STAT1) by phosphorylation in HEL cells and in other model cell lines [2, 8, 12, 38]. The activation of STAT proteins can be monitored by multiple methods. To set up and validate our new screening assays we used the quantitation of STAT5 phosphorylation by Western blot as reference, as it is a robust way to monitor Jak2V617F activity [8]. Here, we describe two new, easy to handle, quick and high throughput compatible intact cell assays to monitor Jak2 activity: (a) a STAT-dependent reporter gene assay, in which Jak2V617F-driven STAT activation leads to expression of the fluorescent ZsGreen reporter and (b) a confocal imaging assay, in which nuclear localization of STAT3-YFP dependent on Jak2V617F activity is monitored. In our comparative study we tested 12 compounds in different assay formats to determine their inhibitory activity towards Jak2V617F and/or to possibly uncover different mechanisms of action, which might ultimately be useful in treatment of MPN. Intriguingly, a first assessment of the compounds using Western blot quantitation of phospho-STAT5 showed that 6 of 12 compounds were not able to inhibit Jak2V617F-mediated signal transduction at nanomolar to low micromolar concentrations. For many drugs low micromolar concentrations can be achieved in patients and interestingly, the low micromolar concentration range is also the limit, for which kinase inhibitors can be assumed to be reasonably specific [39]. JI1, TG101209, TG101348, CYT387, AT9283 and CEP701 all efficiently inhibited Jak2V617F-induced STAT5 phosphorylation with IC_50_ values in the nanomolar concentration range. TG101348 and CYT387 were, however, clearly less active than the other Jak-targeting compounds. All Jak inhibitors also dose dependently suppressed cell growth in HEL cells and in CFC assays with CD34^+^ cells obtained from Jak2V617F-positive MPN patients. None of the remaining compounds (VX680, Sunitinib, WP1066, AG490, LFM-A13 and JNJ7706621) prevented phosphorylation of STAT5 (up to a concentration of 5 μM) and thus none inhibits Jak2V617F in intact cells under these conditions. The failure of AG490 to inhibit Jak (in a different cellular context) was reported by our group before [40]. Interestingly, among the compounds without Jak inhibitory potential, VX680 very efficiently suppressed HEL cell growth, while Sunitinib and WP1066 only had moderate effects. This suggests that combination of VX680 with a Jak inhibitor might have beneficial effects on growth suppression of Jak2V617F-positive cells.

During the analysis of the different inhibitors we compared data from the *in vitro* kinase assays to data obtained in the quantitative cellular assays (quantitative Western blot analysis of STAT5 phosphorylation, reporter gene assay, STAT3-YFP translocation assay). Interestingly, there are more significant differences between the activities of the compounds AT9283, CEP701, JI1, TG101209, TG101348 and CYT387 in intact cells than expected from comparing their behaviour in the *in vitro* kinase assays. These differences may be caused by a number of parameters [39, 41]. However, potent compounds in *in vitro* kinase assays in general also show good cellular activity [39, 41]. Therefore, both technologies should be combined to clearly uncover the activity of a certain compound on its respective target kinases. Unfortunately, it is not possible to extrapolate the exact cellular activity from *in vitro* kinase assay data [39, 42]; this is recapitulated by our comparative analysis (Table 1). (A similar effect is seen when comparing Aurora kinase assays with the corresponding cellular assays). However, active and non-active compounds can be clearly distinguished in the *in vitro* kinase assays. Compounds which did not inhibit Jak2 in *in vitro* kinase assays at single or double digit nanomolar concentrations were inactive in cellular assays (*e.g*. VX680 and Sunitinib). Comparison of our and published *in vitro* kinase assay data (Figure S5B) show that the IC_50_ values do not correspond very well, the greatest divergence of greater than 10-fold is observed for CYT387, which might be owing to differing assay conditions. However, all the efficient Jak2 inhibitors (AT9283, CEP701, JI1, TG101209, TG101348 and CYT387) show low nanomolar activity in our hands and in the published studies.

Another advantage of the cellular assays was the revelation of quantitative and qualitative differences between the Jak-targeting compounds. Interestingly, quantitative evaluation of the phospho-STAT5 Western blot data revealed that TG101209 and CEP701 were most efficiently inhibiting Jak2V617F activity in HEL cells, with IC_50_ values around 100 nM, CEP701 thereby being the most efficient compound among those tested in clinical trials [43]. In our hands, AT9283 [25] was of intermediate potency (IC_50_ around 300 nM) compared with CEP701 and the compounds with less efficacy as TG101348 [44] and CYT387 [45] (IC_50_ values ± 600 nM in HEL cells). A more thorough comparison of the IC_50_ values derived from the different cellular assays (Table 1) revealed that the relative efficiency of JI1, TG101209, TG101348 and CYT387 was always comparable within one assay and that the IC_50_ values for growth inhibition are generally higher than those determined from Western blot experiments tracing STAT5 phosphorylation. However, AT9283 and CEP701 were more efficiently suppressing growth than it would have been expected from comparison with the other Jak-targeting compounds. Such discrepancies can be indicative of a different mode of action of these compounds, as is the case for VX680, which shows no inhibition of Jak2V617F in all assays but suppresses HEL cell growth very efficiently (IC_50_ = 158 nM). The confocal imaging assay revealed an increase in nuclei size after treatment with VX680, AT9283 or CEP701 (Fig. 3A and Figure S6A), whereas JI1, TG101209, TG101348 and CYT387 did not have this effect. The known Aurora kinase inhibitors AT9283 and VX680 [25, 26] showed this effect even below 50 nM, whereas CEP701 increased nuclei size starting at a concentration of 162 nM. CEP701 was, to our knowledge, not reported before to inhibit Aurora kinases in cellular assays, although it was reported to bind Aurora kinases in an *in vitro* kinase binding assay (KINOMEscan) [46]. We confirmed Aurora kinase inhibition by CEP701 using various assays (Fig. 3 and Figure S6). CEP701 and AT9283 show different potencies against Jak2V617F and Aurora kinases. AT9283 seems to inhibit Aurora kinases at very low concentrations (nuclei size increases at concentrations ranging from 6 to 18 nM, in Fig. 3A and Figure S6A), while it inhibits Jak2V617F with an IC_50_ of 309 nM. In contrast, CEP701 is a more potent inhibitor of Jak2V617F (IC_50_ = 97 nM), while Aurora kinase inhibition is efficient only at concentrations from 162 to 486 nM (nuclei size increase in Fig. 3A and Figure S6A). Thus, an advantage of CEP701 might be the fact that it targets the disease-specific Jak2V617F protein more efficiently than it inhibits Aurora kinases. This might lead to fewer side effects than compounds whose main target is not Jak2V617F (*e.g*. AT9283).

When testing the compounds in the CFC assay with haematopoietic progenitor cells (CD34^+^) derived from Jak2V617F-positive MPN patients, we found that in general Jak inhibitors preferentially suppressed growth of erythroid colonies, whereas Aurora kinase inhibitors (AT9283, VX680, AZD1152, Danusertib and SNS314) preferentially blocked myeloid colony growth (Fig. 4 and Figure S7). AT9283 was so efficient in suppressing colony growth at very low concentrations, that it is questionable whether Jak2V617F inhibition plays any role in this effect at all. Interestingly, CEP701 showed an intermediate behaviour compared with the Jak or the Aurora kinase inhibitors. Myeloid colony suppression was equal or slightly better compared with erythroid colony suppression at a given concentration of CEP701. As MPN patients also have significant numbers of myeloid inflammatory cells, the Aurora kinase-inhibitory potential of CEP701 might turn out to be especially interesting in suppressing the disease-associated inflammation [47]. The effect of CEP701 (Jak and Aurora kinase inhibition) can be imitated using two inhibitors, one with predominant Jak inhibitory activity (TG101209) and one with predominant Aurora kinase-inhibitory activity (VX680). Indeed, combination of VX680 and TG101209 (Fig. 5) decreased the number of erythroid and myeloid colonies in parallel reflecting the beneficial effects of both inhibitors. Interestingly, we could demonstrate a synergistic effect of the combined treatment with VX680 and TG101209 in cell lines expressing Jak2V617F emphasizing that the combination of a Jak and an Aurora kinase inhibitor is more efficient in suppressing cell proliferation than the respective single treatment. We conclude that Jak2V617F and Aurora kinase inhibition if correctly modulated (as in CEP701) is an attractive concept to target erythroid growth and the myeloid cell-governed inflammation occurring in MPN. An alternative approach might be to combine two compounds for the treatment as long as the side effects caused by the compounds remain tolerable.

Clinical trials involving the Aurora kinase inhibitors AT9283 and VX680 for the treatment of MPN or myeloid leukaemia have been terminated (http://www.clinicaltrials.gov). However, other Aurora kinase inhibitors (AZD1152 and Danusertib) are tested in clinical trials for application in acute myeloid leukaemia or relapsed chronic myeloid leukaemia and might prove to be well tolerable. Interesting Jak inhibitors to test in conjunction with Aurora kinase inhibitors are those which have entered clinical trials for MPN or other haematologic malignancies (TG101348, LY2784544, CYT387, SB1518) [15] and INCB018424, which has been approved for treatment of myelofibrosis by the FDA and the European Commission. Jak inhibitors in clinical trials for rheumatoid arthritis or other autoimmune disorders [Tofacitinib (CP690,550), LY3009104, GLPG0634, VX509 or AC430] [15] might also be used in conjunction with low dose Aurora kinase inhibitors to control inflammation.

## Supporting information

**Figure S1** (A) Chemical structures of compounds, which have Jak inhibitory activity in living cells at concentrations below or around 1 μM. (B) Structures of compounds, which had no potential to inhibit Jak2V617F-dependent signal transduction below a concentration of 5 μM.

**Figure S2** Validation of effects of the investigated compounds in other relevant cell systems.

**Figure S3** Characterization of the HEK-V617F-STAT-Rep. cells.

**Figure S4** Characterization of the γ2A-FRT-TI-Jak2V617F/STAT3-YFP cells.

**Figure S5** *In vitro* kinase data.

**Figure S6** CEP701 has Aurora kinase inhibitory activity.

**Figure S7** Aurora kinase inhibition rather suppresses growth of myeloid colonies.

**Figure S8** Jak and Aurora kinase inhibitors cooperate to suppress growth of Jak2V617F-expressing cells.

**Figure S9** Chou-Talalay analysis: TG101209 and VX680 synergistically inhibit proliferation of BA/F3-EpoR-Jak2V617F cells.
