# Supplementary material for: Cooperative effects of Janus and Aurora kinase inhibition by CEP701 in cells expressing Jak2V617F
Source: J Cell Mol Med. 2013 Jan 10;17(2):265–76. doi: 10.1111/jcmm.12005 (PMC3822589; doi:10.1111/jcmm.12005)
Supplement: Supplementary file 1 [file jcmm0017-0265-SD1.pdf]

Materials and methods

Cell culture

The Jak2V617F-positive UKE-1 cell line was a gift from Dr. Walter Fiedler (University Medical Center Hamburg-Eppendorf, Hamburg, Germany). The cells were cultured in Iscove's modified Dulbecco's medium (IMDM, Gibco, Life Technologies, Carlsbad, CA, USA) supplemented with 10% FBS, 100 mg/l streptomycin, 60 mg/l penicillin, 10 ng/ml IL-3 and 10 ng/ml GM-CSF. The BA/F3-EpoR-Jak2V617F cells (a murine pro B cell line, stably expressing Jak2V617F and the Epo receptor) were a kind gift from Dr. Thomas Fischer (Otto-von-Guericke-University, Magdeburg, Germany). They were maintained in RPMI-1640 medium (Lonza, Basel, Switzerland) supplemented with 10% FBS, 100 mg/l streptomycin, 60 mg/l penicillin, 2 mM L-glutamine, 2 μg/ml puromycin and 1 mg/ml G418.

The CMK (acute megakaryocytic leukaemia cells expressing Jak3A572V) and K-562 (chronic myeloid leukaemia cells positive for Bcr-Abl) cell lines were cultured in RPMI-1640 medium supplemented with 10% FBS, 100 mg/l streptomycin, 60 mg/l penicillin and 2 mM L-glutamine. The colon carcinoma cell line HCT-116 and the fibrosarcoma cell line 2C4 were maintained in Dulbecco's modified Eagle's medium (DMEM, Lonza) supplemented with 10% FBS, 100 mg/l streptomycin, 60 mg/l penicillin and 25 mM HEPES.

Generation of cDNA constructs

Standard cloning procedures were followed throughout. The integrity of all constructs was verified by DNA sequencing at GATC Biotech (Konstanz, Germany). Restriction enzymes were from New England Biolabs (Ipswich, MA, USA).

pSTAT-ZsGreen was generated as follows: pZsGreen1-DR was purchased from Clontech Laboratories (Mountain View, CA, USA). A new multiple cloning site with PacI, AgeI, NheI, EcoRI, AscI restriction sites and a TATA box were introduced by opening the vector with XhoI and SmaI and inserting the annealed oligonucleotides 5'-TCGAGAGCTTAATTAAAGCACCG GTAAAGCTAGCTAGAATTCGAAGGCGCGCCTATATAACTGGTACCACAGGGCCC-3' and 5'-GGGCCCTGTGGTACCAGTTATATAGGCGCGCCTTCGAATTCTTAGCTAGCTTT ACCGGTGCTTTAATTAAGCTC-3'. 12 STAT binding sites were then introduced in two steps. First, the annealed oligonucleotides 5'-CCGGTTTCCGGGAAAGCGTTTCCGGGAAAGCGTT TCCGGGAAAGCGTTTCCGGGAAAGCTTTCCCGTAAAGCTTTCCCGTAAAG-3' and 5'-CTAGCTTTACGGGAAAGCTTTACGGGAAAGCTTTCCCGGAAACGCTTTCCCGGAA ACGCTTTCCCGGAAACGCTTTCCCGGAAA-3' were inserted using AgeI and NheI. Then, the annealed oligonucleotides 5'-CTAGCGGTTTCCGGGAAAGCGTTTCCGGGAAAGCGTT TCCGGGAAAGCGTTTCCGGGAAAGCTTTCCCGTAAAGCTTTCCCGTAAAG-3' and 5'-AATTCTTTACGGGAAAGCTTTACGGGAAAGCTTTCCCGGAAACGCTTTCCCGGAA ACGCTTTCCCGGAAACGCTTTCCCGGAAACCG-3' were inserted using NheI and EcoRI yielding a reporter gene construct with 8 STAT5 consensus binding sites from the CIS promoter [1] and 4 STAT3/1 consensus binding sites of the mutated SIE sequence (m67SIE) [2-3] preceding the ZsGreen-coding region.

The plasmid pBOF/V5-Jak2V617F/STAT3-YFP was built as follows: The generation of the inducible, bidirectional Flp-in vector pBOF was described before [4]. STAT3-YFP cDNA was sub-cloned into pBOF using SbfI and SwaI restriction sites. V5-Jak2V617F was then sub-cloned using AgeI and NheI.

pTET-on$_{puro}$ was derived from the vector pTET-on (Clontech Laboratories) by replacing the neomycin resistance gene by a puromycin resistance gene, which was amplified by PCR and inserted into BglII and BstBI restriction sites.

Generation of stable cell lines

γ2A cells (a Jak2-deficient fibrosarcoma cell line) were a kind gift from I. M. Kerr. The cells were cultured in Dulbecco's modified Eagle's medium (DMEM, Lonza) supplemented with 10% FBS, 100 mg/l streptomycin, 60 mg/l penicillin and 25 mM HEPES. The cells were transfected with Superfect (Qiagen, Hilden, Germany) or Lipofectamine 2000 (Invitrogen, Life Technologies, Carlsbad, CA, USA) according to protocol. γ2A cells were first transfected with the plasmid pFRT-lac-zeo (Invitrogen, Life Technologies) (FRT: FLP Recombination Target) and selected for stable integration using 100 µg/ml Zeocin (InvivoGen, San Diego, CA, USA) to generate γ2A-FRT cells. Clones were checked for the presence of the "Flp-in" site using the β-gal staining kit (Invitrogen, Life Technologies). The γ2A-FRT cells were then transfected with pTET-on$_{puro}$ and selected for stable integration using 1 µg/ml puromycin (InvivoGen). Clones were tested for the presence of the reverse tetracycline-responsive transcriptional activator by PCR. The resulting γ2A-FRT-TI (TI: Tet-Inducible) cells were transfected with pBOF/V5-Jak2V617F/STAT3-YFP and selected for stable integration using hygromycin (InvivoGen) to yield the resulting γ2A-FRT-TI-Jak2V617F/STAT3-YFP. Cells were controlled for the expression of STAT3-YFP by fluorescence microscopy after induction with 5 µg/ml doxycycline for 24 hrs.

HEK293-FRT-TO-EpoR-Jak2V617F cells [5] were maintained in Dulbecco's modified Eagle's medium (DMEM, Lonza) supplemented with 10% FBS, 100 mg/l streptomycin, 60 mg/l

penicillin and 25 mM HEPES. The cells were transfected with the pSTAT-ZsGreen reporter plasmid using *Trans*IT-LT1 (Mirus Bio, Madison, WI, USA) and were selected for stable integration using 1 mg/ml G418 (InvivoGen). Clones were tested for expression of the ZsGreen protein by fluorescence microscopy after inducing Jak2V617F expression with 5 ng/ml doxycycline for 24 hrs.

Quantification of STAT5 phosphorylation

The quantification of the phospho-STAT5 signals was performed using the analysis software V3.0 provided by LI-COR Biosciences (Lincoln, NE, USA). The phospho-STAT5 signal was normalized with respect to the signal of total STAT5 (loading control) for each lane. Each normalized phospho-STAT5 signal was then divided by the mean of all normalized phospho-STAT5 signals of one membrane to adjust for the possible variation of signal intensity between different membranes (each membrane includes one biological replicate). The signal intensity of phospho-STAT5 is represented as percentage of the strongest signal.

CyQUANT® Cell Proliferation Assay

The cells were cultured with inhibitor at indicated concentrations or left untreated for up to 72 hrs. Afterwards, they were incubated with the freshly prepared detection reagent of the CyQUANT® Direct Cell Proliferation Assay Kit (Invitrogen, Life Technologies) according to the manufacturer's instructions. The fluorescence signal was detected with λ_{exc} = 485 nm and λ_{em} = 520 nm in a FLUOstar OPTIMA microplate reader (BMG LABTECH, Ortenberg, Germany). The amount of inhibitor-treated cells was calculated as percentage of maximum number of cells (= untreated control) from biological replicates (n = 3 - 5).

Analysis of drug combinations according to the Chou-Talalay method

The growth inhibitory effect of two single drugs (VX680 and TG101209) and their combination was assessed by the CyQUANT® Direct Cell Proliferation Assay Kit (see above). The compounds were applied in various concentrations while keeping the concentration ratio between the two inhibitors constant (for details please see legend for Fig. S9). A dose-effect analysis of the drug combination was performed using the CompuSyn software [6-7] according to the Chou-Talalay method [8]. The Combination Index (CI) value is then the quantitative measure of the dose-effect analysis of a drug combination, meaning CI < 1 represents synergism, CI > 1 corresponds to antagonism and CI ≈ 1 represents an additive effect for the drug combination.

In vitro *kinase assays*

The Jak2, Aurora A and Aurora B kinase assays were performed by Caliper Discovery Alliances & Services "CDAS" using the LabChip technology from Caliper Life Sciences (Hopkinton, MA, USA) using standard conditions and Staurosporine as a reference compound. Ten half log dilutions of the different compounds were used. All assays were performed at K_m^{ATP} for each kinase. Briefly, the Jak2 kinase assays were performed using 15 nM of enzyme, 1.5 µM of substrate and 14 µM of ATP in 100 mM HEPES, pH 8.0 supplemented with 0.004% Triton X-100. The Aurora A kinase assays were performed using 4 nM of enzyme, 1.5 µM of substrate and 25 µM of ATP in 100 mM HEPES, pH 7.5 supplemented with 0.1% Brij-35. The Aurora B kinase assays were performed using 5 nM of enzyme, 1.5 µM of substrate and 5 µM of ATP in 100 mM HEPES, pH 7.5 supplemented with 0.1% Brij-35. The project was completed using the Caliper LabChip 3000 and a 12-sipper LabChip. IC_{50} values were determined using GraphPad Prism 5.01, log [inhibitor] vs. response-Variable slope 4PL curve fit from duplicate experiments.

Cell size determination

HEL cells were cultivated with 600 nM of CEP701, TG101209, VX680 or 100 nM of AT9283 or left untreated for 24, 48 or 72 hrs. The average cell diameter (d) of HEL cells was determined with a CEDEX XS cell counter (Roche Diagnostics, Basel, Switzerland). The cell volume (V) was then calculated using the formula: $(4/3) \cdot \pi \cdot (d/2)$.

References

1. **Matsumoto A, Masuhara M, Mitsui K, *et al.*** CIS, a cytokine inducible SH2 protein, is a target of the JAK-STAT5 pathway and modulates STAT5 activation. *Blood*. 1997; 89: 3148-54.

2. **Wagner BJ, Hayes TE, Hoban CJ, Cochran BH**. The SIF binding element confers sis/PDGF inducibility onto the c-fos promoter. *Embo J*. 1990; 9: 4477-84.

3. **Haan S, Keller JF, Behrmann I, *et al.*** Multiple reasons for an inefficient STAT1 response upon IL-6-type cytokine stimulation. *Cell Signal*. 2005; 17: 1542-50.

4. **Haan C, Rolvering C, Raulf F, *et al.*** Jak1 has a dominant role over Jak3 in signal transduction through gammac-containing cytokine receptors. *Chem Biol*. 2011; 18: 314-23.

5. **Haan S, Wuller S, Kaczor J, *et al.*** SOCS-mediated downregulation of mutant Jak2 (V617F, T875N and K539L) counteracts cytokine-independent signaling. *Oncogene*. 2009; 28: 3069-80.

6. **Chou TC, Martin N**. CompuSyn for Drug Combinations and for General Dose-Effect Analysis, Software and User's Guide: A Computer Program for Quantitation of Synergism and Antagonism in Drug Combinations, and the Determination of IC50 and ED50 and LD50 Values. *ComboSyn Inc, Paramus, NJ, USA*. 2005; www.combosyn.com.

7. **Chou TC**. Theoretical basis, experimental design, and computerized simulation of synergism and antagonism in drug combination studies. *Pharmacol Rev*. 2006; 58: 621-81.

8. **Chou TC, Talalay P**. Quantitative analysis of dose-effect relationships: the combined effects of multiple drugs or enzyme inhibitors. *Adv Enzyme Regul*. 1984; 22: 27-55.

9. **Howard S, Berdini V, Boulstridge JA, *et al.*** Fragment-based discovery of the pyrazol-4-yl urea (AT9283), a multitargeted kinase inhibitor with potent aurora kinase activity. *J Med Chem*. 2009; 52: 379-88.

10. **Hexner EO, Serdikoff C, Jan M, *et al.*** Lestaurtinib (CEP701) is a JAK2 inhibitor that suppresses JAK2/STAT5 signaling and the proliferation of primary erythroid cells from patients with myeloproliferative disorders. *Blood*. 2008; 111: 5663-71.

11. **Burns CJ, Bourke DG, Andrau L, *et al.*** Phenylaminopyrimidines as inhibitors of Janus kinases (JAKs). *Bioorg Med Chem Lett*. 2009; 19: 5887-92.

12. **Thompson JE, Cubbon RM, Cummings RT, *et al.*** Photochemical preparation of a pyridone containing tetracycle: a Jak protein kinase inhibitor. *Bioorg Med Chem Lett*. 2002; 12: 1219-23.

13. **Fridman J, Wen X, Caulder E, *et al.*** Abstract #4727: Resistance to tyrosine kinase inhibitors reversed by selective JAK inhibition. *AACR Meeting Abstracts*. 2009; 4727.

14. **Pardanani A, Hood J, Lasho T, *et al.*** TG101209, a small molecule JAK2-selective kinase inhibitor potently inhibits myeloproliferative disorder-associated JAK2V617F and MPLW515L/K mutations. *Leukemia*. 2007; 21: 1658-68.

15. **Wernig G, Kharas MG, Okabe R, *et al.*** Efficacy of TG101348, a selective JAK2 inhibitor, in treatment of a murine model of JAK2V617F-induced polycythemia vera. *Cancer Cell*. 2008; 13: 311-20.

16. **Karaman MW, Herrgard S, Treiber DK, *et al.*** A quantitative analysis of kinase inhibitor selectivity. *Nat Biotechnol*. 2008; 26: 127-32.

17. **Giles F, Bergstrom DA, Garcia-Manero G, *et al.*** MK-0457 is a novel aurora kinase and janus kinase 2 (JAK2) inhibitor with activity in transformed JAK2-Positive myeloproliferative disease (MPD). *Blood*. 2006; 108: 4893.

Figures

Figure S1

A

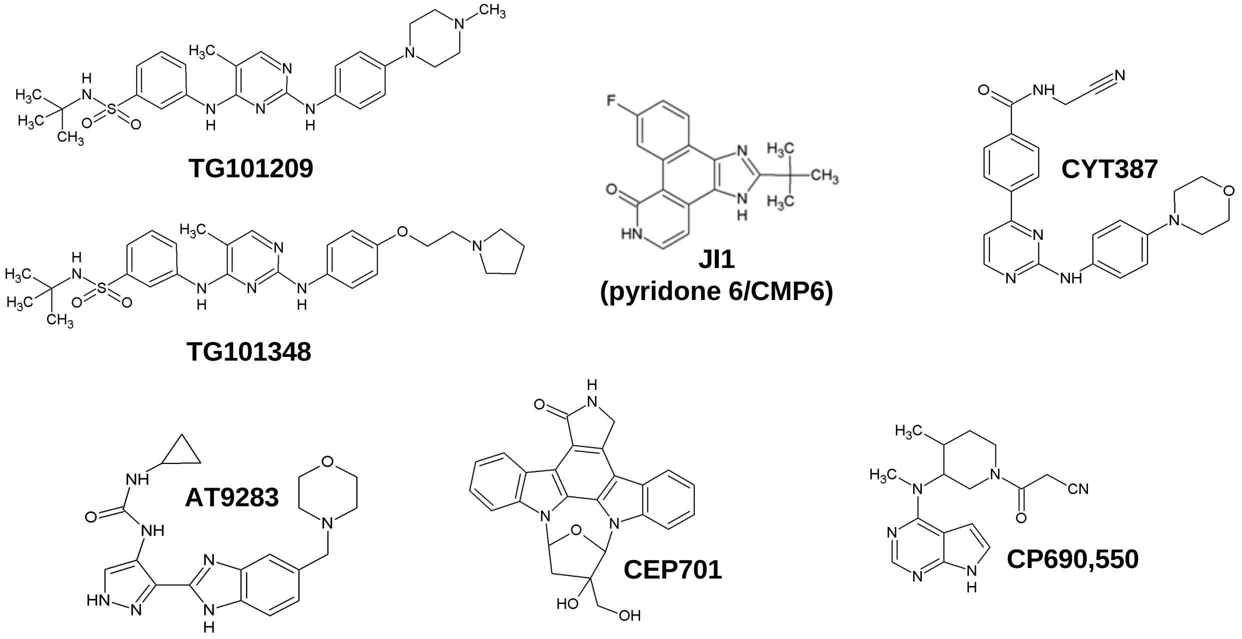

B

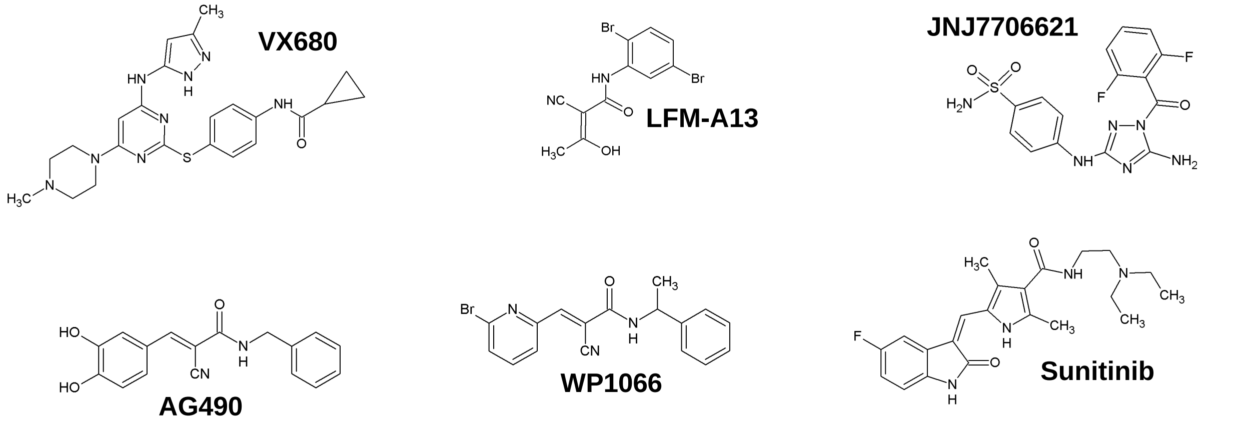

A, Chemical structures of compounds, which have Jak inhibitory activity in living cells at concentrations below or around 1 μM. B, Structures of compounds, which had no potential to inhibit Jak2V617F-dependent signal transduction below a concentration of 5 μM.

Figure S2

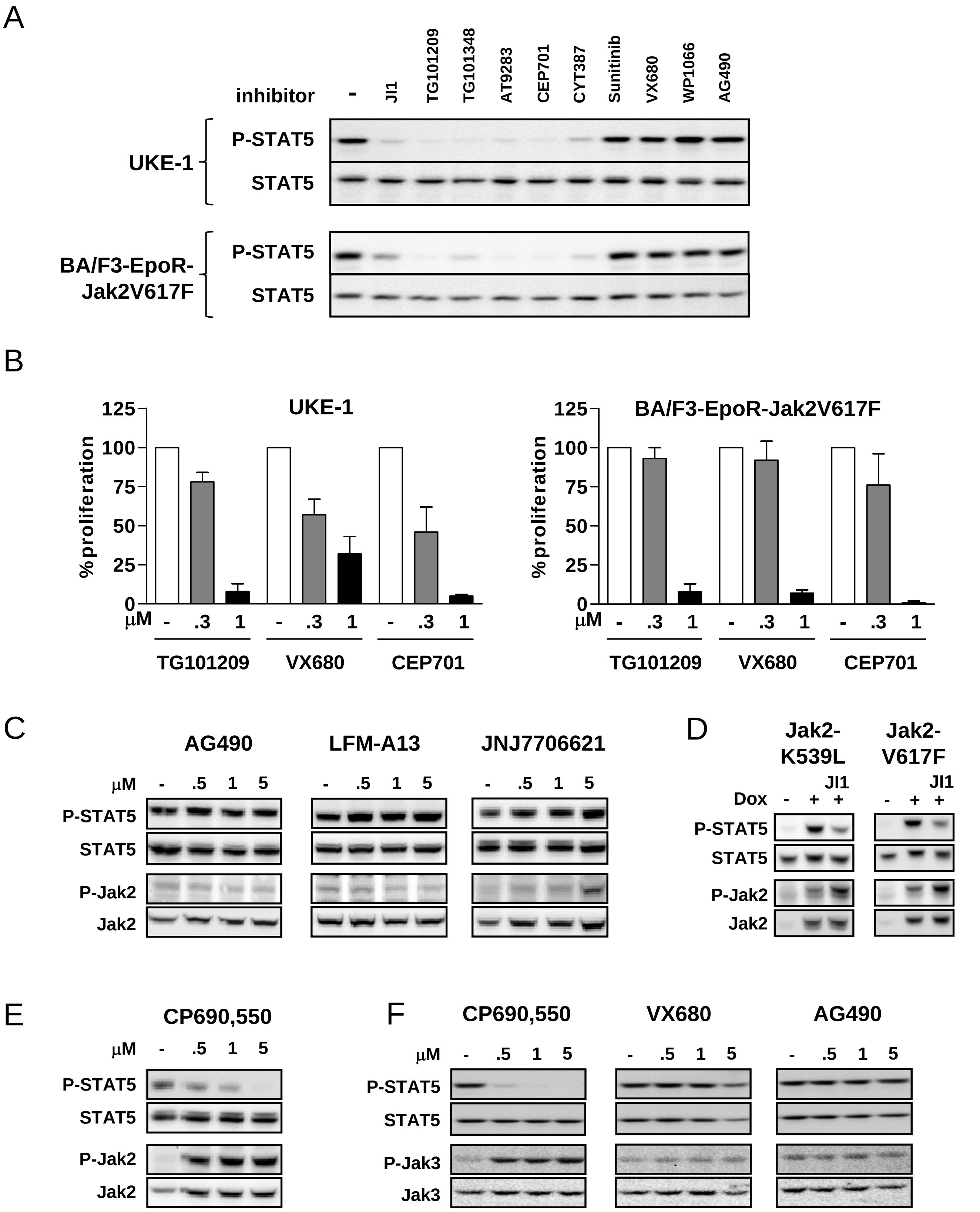
A
inhibitor
-
JI1
TG101209
TG101348
AT9283
CEP701
CYT387
Sunitinib
VX680
WP1066
AG490
UKE-1
P-STAT5
STAT5
BA/F3-EpoR-Jak2V617F
P-STAT5
STAT5
B
UKE-1
BA/F3-EpoR-Jak2V617F
%proliferation
125
100
75
50
25
0
μM
- .3 1 - .3 1 - .3 1
TG101209
VX680
CEP701
C
AG490
LFM-A13
JNJ7706621
μM - .5 1 5
P-STAT5
STAT5
P-Jak2
Jak2
D
Jak2-K539L
Jak2-V617F
JI1
Dox - + +
P-STAT5
STAT5
P-Jak2
Jak2
E
CP690,550
μM - .5 1 5
P-STAT5
STAT5
P-Jak2
Jak2
F
CP690,550
VX680
AG490
μM - .5 1 5
P-STAT5
STAT5
P-Jak3
Jak3

Figure S2: Validation of effects of the investigated compounds in other relevant cell systems

A, UKE-1 and BA/F3-EpoR-Jak2V617F cells were treated with different inhibitors at a concentration of 2.5 μM or left untreated for 3 hrs. The expression and phosphorylation state of STAT5 (P-STAT5) was assessed by Western blot immunodetection. B, UKE-1 and BA/F3-EpoR-Jak2V617F cells were treated with TG101209, VX680 and CEP701 at concentrations of 0.3 or 1 μM or left untreated for 72 hrs. Cell proliferation was assessed by the CyQUANT cell proliferation assay. C, HEL cells were treated with 0.5, 1 or 5 μM of the indicated inhibitors or left untreated for 3 hrs. The expression and phosphorylation state of STAT5 (P-STAT5) and Jak2 (P-Jak2) was assessed by Western blot immunodetection. D, HEK-EpoR cells stably and inducibly expressing Jak2V617F or Jak2K539L were treated with 1 ng/ml doxycycline (+) or left untreated (-) for 12 hrs. Subsequently, the cells were treated with 1 μM of JI1 for 3 hrs as indicated. Western blots were detected as described in C. E, HEL cells were treated with 0.5, 1 or 5 μM of CP690,550 or left untreated for 3 hrs. Western blots were detected as described in C. F, CMK cells were treated with 0.5, 1 or 5 μM of the indicated inhibitors or left untreated for 3 hrs. The expression and phosphorylation state of STAT5 (P-STAT5) was assessed by Western blot immunodetection. To assess the phosphorylation state of Jak3 (P-Jak3) an anti-phospho-Jak1 antibody, which is cross-reactive and also detects phospho-Jak3 [4], was used.

Figure S3

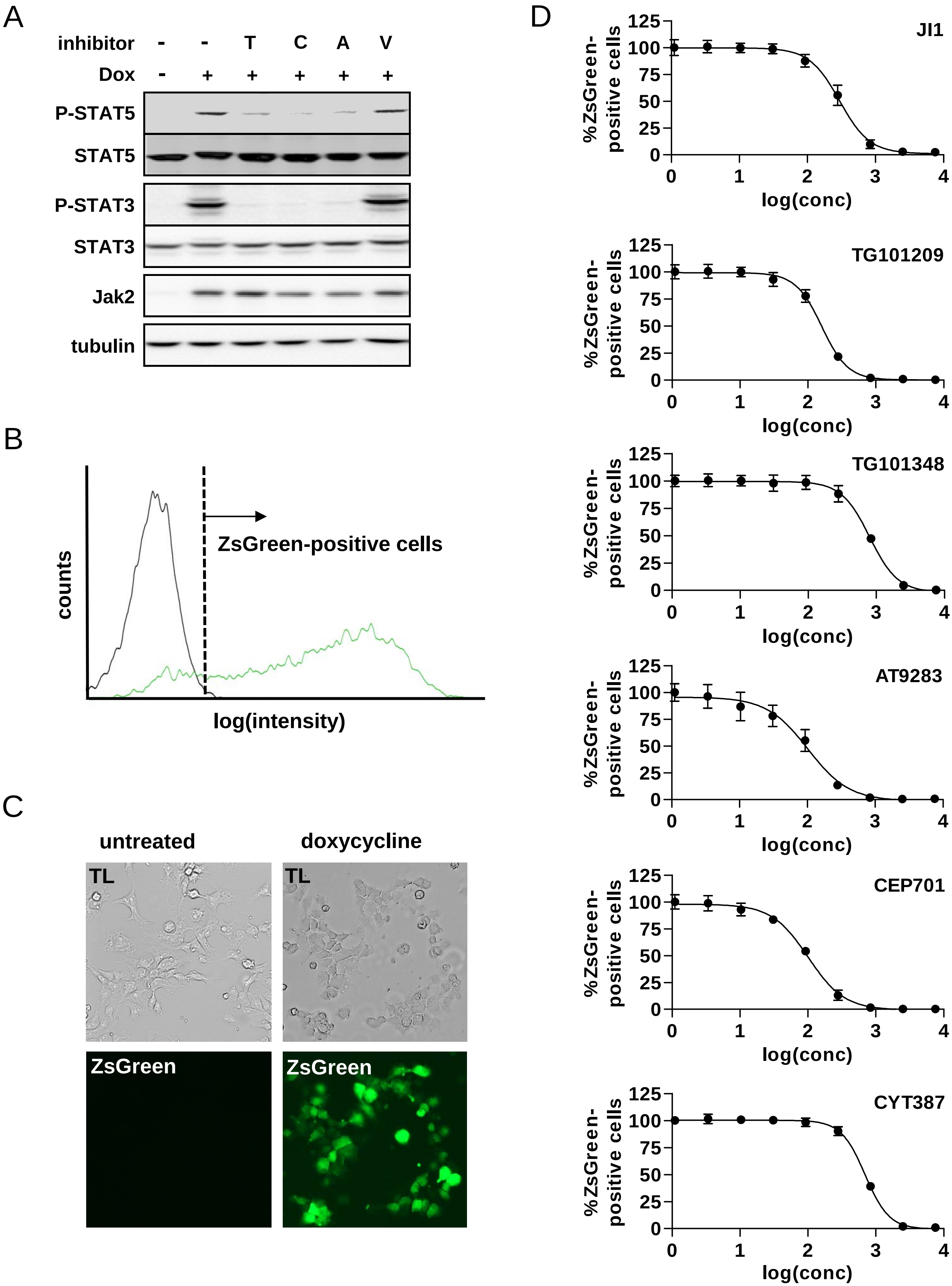
A
inhibitor - - T C A V
Dox - + + + + +
P-STAT5
STAT5
P-STAT3
STAT3
Jak2
tubulin
B
counts
ZsGreen-positive cells
log(intensity)
C
untreated
doxycycline
TL
TL
ZsGreen
ZsGreen
D
JI1
TG101209
TG101348
AT9283
CEP701
CYT387
%ZsGreen-positive cells
log(conc)
125
100
75
50
25
0
0
1
2
3
4

Figure S3: Characterization of the HEK-V617F-STAT-Rep. cells

A, HEK-V617F-STAT-Rep. cells were treated with 10 ng/ml doxycycline for 12 hrs. Subsequently, TG101209 (T), CEP701 (C), AT9283 (A) or VX680 (V) was added at a concentration of 2.5 μM for 3 hrs. The phosphorylation state of STAT5 (P-STAT5) and STAT3 (P-STAT3) and the expression of STAT5, STAT3, Jak2 and tubulin was assessed by Western blot immunodetection. B, HEK-V617F-STAT-Rep. cells were treated with 10 ng/ml doxycycline (green line) or left untreated (black line) for 24 hrs and were then subjected to flow cytometric analysis. ZsGreen-positive cells were gated so that less than 1% of the untreated cells were considered ZsGreen-positive. In the histogram all events to the right of the dashed line are considered cells expressing ZsGreen. C, HEK-V617F-STAT-Rep. cells were treated with 10 ng/ml doxycycline (right panel) or left untreated (left panel) for 24 hrs. Induction with doxycycline leads to green-fluorescent cells compared to untreated cells not showing any green fluorescence. D, Determination of the IC_{50} values of the different inhibitors in the reporter gene assay by flow cytometry: HEK-V617F-STAT-Rep. cells were induced with 10 ng/ml doxycycline and were additionally treated with 7500, 2500, 833, 278, 93, 31, 10.3, 3.4 or 1.1 nM of the different inhibitors for 24 hrs. IC_{50} values were determined using GraphPad Prism 5.01, log [inhibitor] vs. response-Variable slope 4PL curve fit from duplicate experiments. The graphs shows mean values ± standard deviation of three biological replicate experiments, from which the IC_{50} values were determined.

Figure S4

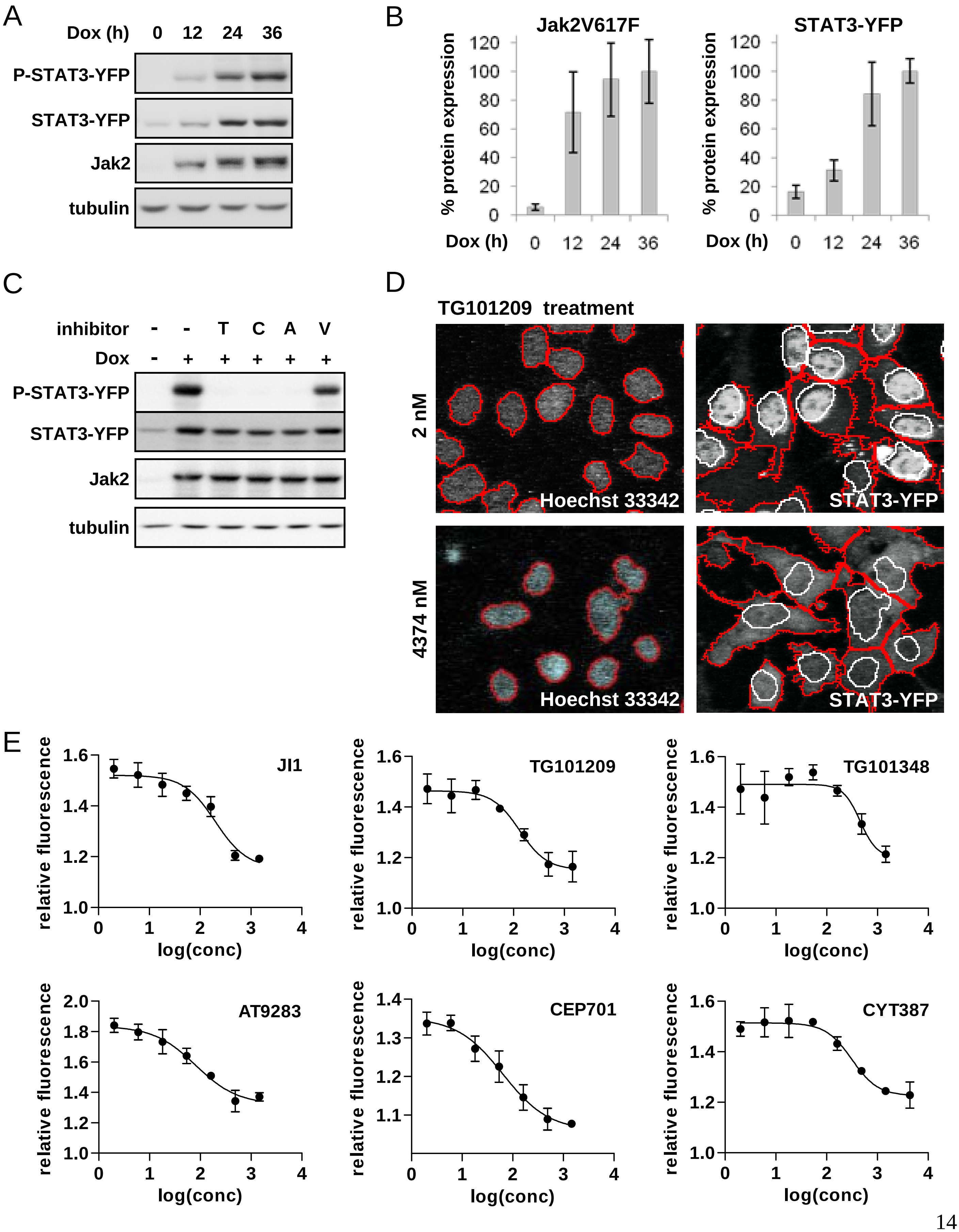

A
Dox (h) 0 12 24 36
P-STAT3-YFP
STAT3-YFP
Jak2
tubulin
B
Jak2V617F
STAT3-YFP
% protein expression
Dox (h) 0 12 24 36
C
inhibitor - - T C A V
Dox - + + + + +
P-STAT3-YFP
STAT3-YFP
Jak2
tubulin
D
TG101209 treatment
2 nM
4374 nM
Hoechst 33342
STAT3-YFP
E
JI1
TG101209
TG101348
AT9283
CEP701
CYT387
relative fluorescence
log(conc)

Figure S4: Characterization of the γ2A-FRT-TI-Jak2V617F/STAT3-YFP cells

A, γ2A-FRT-TI-Jak2V617F/STAT3-YFP cells were induced with 5 μg/ml doxycycline for different time points (as indicated). Quantitative Western blot detection was performed as described in Materials and Methods. The expression kinetics of STAT3-YFP and Jak2V617F upon doxycycline treatment was assessed by Western blot immunodetection with anti-GFP and anti-Jak2 antibodies, respectively. Phosphorylation of STAT3-YFP by Jak2V617F was detected with an anti-phospho-STAT3 antibody. Tubulin detection was used to show equal loading. B, Quantitation of triplicate experiments (similar to A) of Jak2V617F and STAT3-YFP expression is shown. After 24 hrs both proteins are robustly expressed. Therefore, this time point was chosen to perform the assay. C, γ2A-FRT-TI-Jak2V617F/STAT3-YFP cells were treated with 5 μg/ml doxycycline for 24 hrs. Subsequently, TG101209 (T), CEP701 (C), AT9283 (A) or VX680 (V) at a concentration of 2.5 μM was added for 3 hrs. The expression and phosphorylation state of STAT3-YFP (P-STAT3-YFP) and the expression of Jak2 and tubulin were assessed by Western blot immunodetection. D, After induction with doxycycline γ2A-FRT-TI-Jak2V617F/STAT3-YFP cells were treated with 2 or 4374 nM of TG101209 for 12 hrs. The left panel shows the automatic recognition of the nuclei by the "Cell Profiler" software; nuclear borders are highlighted by a red line. The right panel shows the automatic recognition of the YFP signal by the software, also delineated by a red line. The nuclear borders are additionally depicted by a white line in these pictures. When Jak2V617F is not inhibited at low concentrations of TG101209 (2 nM), STAT3-YFP is located mostly in the nucleus. Upon inhibition of Jak2V617F (4374 nM TG101209) STAT3-YFP is localized rather in the cytoplasm. E, Determination of the IC_{50} values of the different inhibitors in the STAT3-YFP translocation assay: γ2A-FRT-TI-Jak2V617F/STAT3-YFP were induced with 5 μg/ml doxycycline and were additionally treated with 7500, 2500, 833, 278, 93, 31, 10.3, 3.4 or 1.1 nM of the different inhibitors for 12 hrs. IC_{50} values were determined using GraphPad Prism 5.01, log [inhibitor] vs. response-Variable slope 4PL curve fit from duplicate experiments. The graphs show the curves from 4 technical replicates of a representative biological replicate.

Figure S5

A

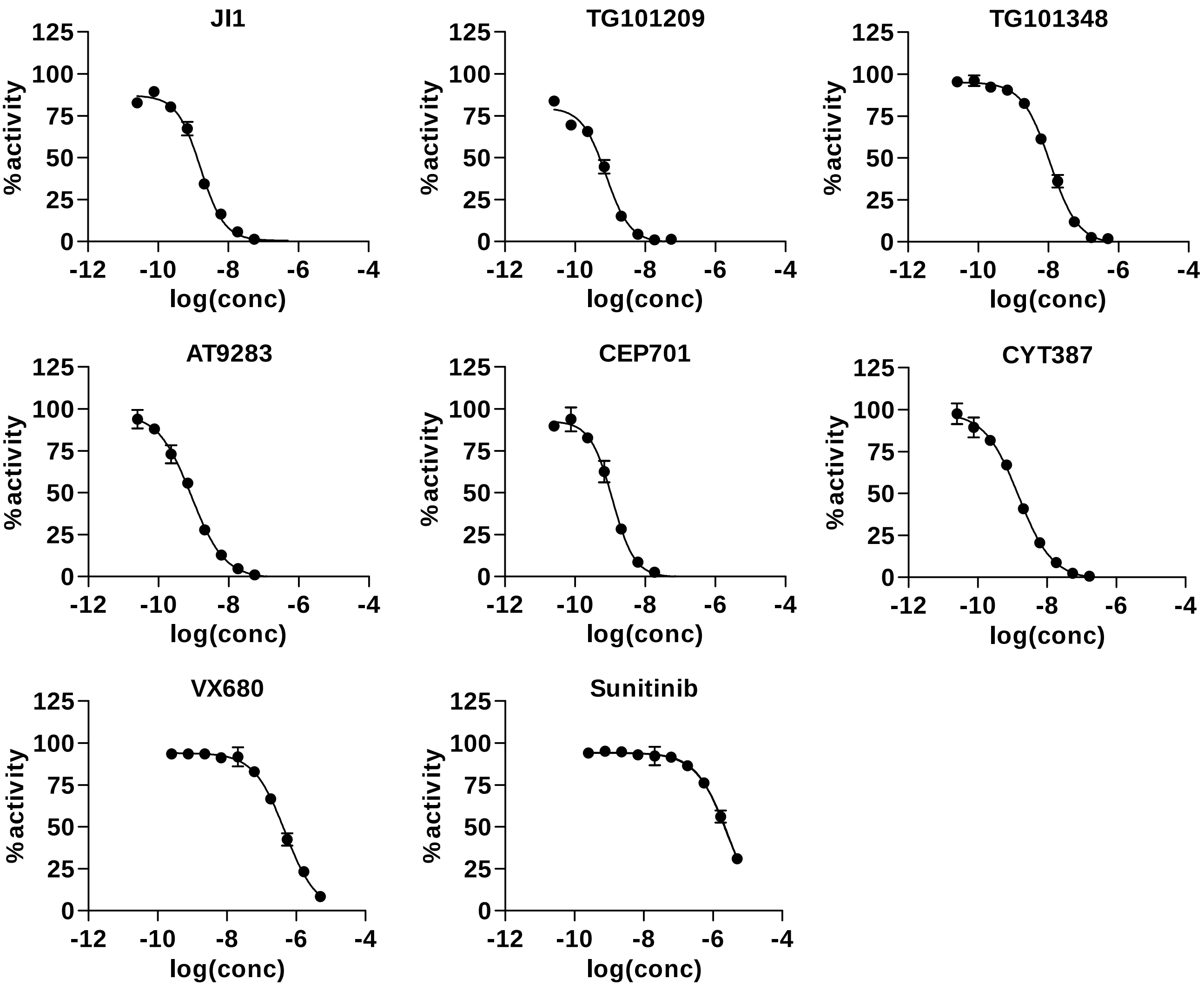

B

Compound	Caliper (nM)	Literature (nM)	Reference
**AT9283**	0.88	1.2	[9]
**CEP701**	1.2	0.9	[10]
**CYT387**	1.5	18	[11]
**JI1 (pyridone 6)**	1.6	2.1	[4, 12-13]
**TG101209**	0.76	6	[14]
**TG101348**	11	3	[15]
**Sunitinib**	2400	410[a]	[16]
**VX680**	480	190[a]<br>123 (WT)<br>295 (V617F)	[16-17]

[a] IC_{50} value was determined with a competitive binding assay (KINOMEscan).

Figure S5: **In vitro *kinase data***

A, IC_{50} values for Jak2 inhibition were determined in an *in vitro* kinase assay for the indicated inhibitors. The assays were performed by Caliper Discovery Alliances & Services "CDAS" applying the LabChip technology from Caliper Life Sciences using standard conditions and Staurosporine as a reference compound. IC_{50} values were determined using GraphPad Prism 5.01, log [inhibitor] vs. response-Variable slope 4PL curve fit from duplicate experiments. B, The table shows IC_{50} values of *in vitro* Jak2 kinase assays determined by Caliper Life Sciences in comparison to data found in the literature.

Figure S6A-D

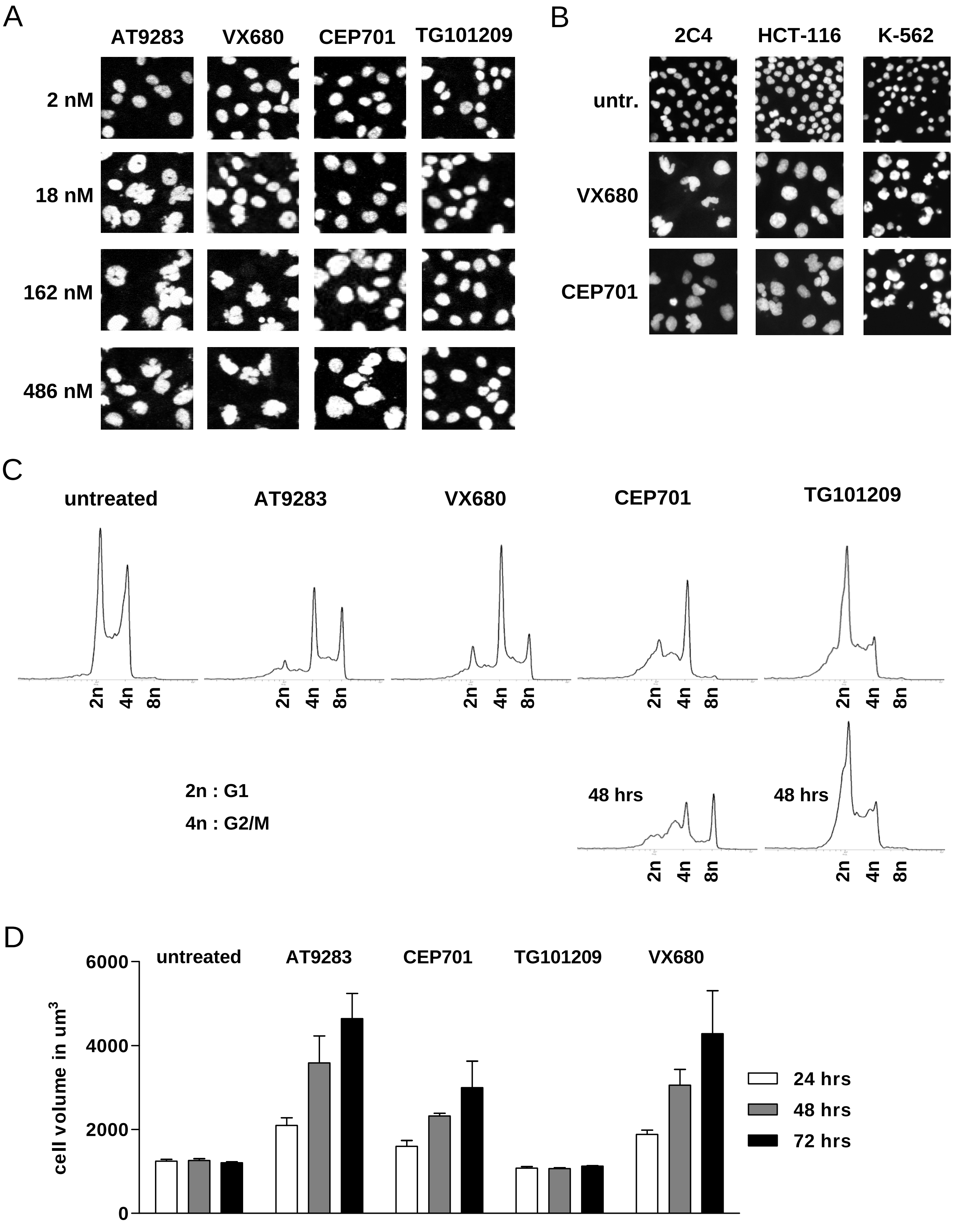
A
AT9283
VX680
CEP701
TG101209
2 nM
18 nM
162 nM
486 nM
B
2C4
HCT-116
K-562
untr.
VX680
CEP701
C
untreated
AT9283
VX680
CEP701
TG101209
2n
4n
8n
48 hrs
48 hrs
2n : G1
4n : G2/M
D
untreated
AT9283
CEP701
TG101209
VX680
cell volume in um³
6000
4000
2000
0
24 hrs
48 hrs
72 hrs

Figure S6A-D: CEP701 has Aurora kinase inhibitory activity.

A, γ2A-FRT-TI-Jak2V617F/STAT3-YFP cells were treated with the indicated inhibitors for 24 hrs. Subsequently, the cells were stained with the DNA dye Hoechst 33342 at a concentration of 1 µg/ml for 20 min. Confocal imaging pictures show the changes of nuclear appearance. B, 2C4, HCT-116, and K-562 cells were treated with 500 nM of VX680 or CEP701 or left untreated for 48 hrs. Subsequently, the nuclei were stained with Hoechst 33342 at a concentration of 1 µg/ml for 20 min. C, HEL cells were treated with 500 nM CEP701, VX680, TG101209 or 100 nM AT9283 for 24 or 48 hrs and then subjected to cell cycle analysis. The histograms demonstrate the emergence of an 8n peak after 24 hrs of treatment with AT9283 and VX680 and after 48 hrs of CEP701 treatment. Cell cycle profiles of TG101209 treated cells are shown as negative control. The results of one representative technical replicate of three biological replicates are shown. D, HEL cells were treated with 600 nM CEP701, TG101209, VX680 or 100 nM AT9283 or left untreated for 24, 48 or 72 hrs. The mean values of the cell volume with standard deviations from 3 biological triplicates are shown.

Figure S6E-F

E

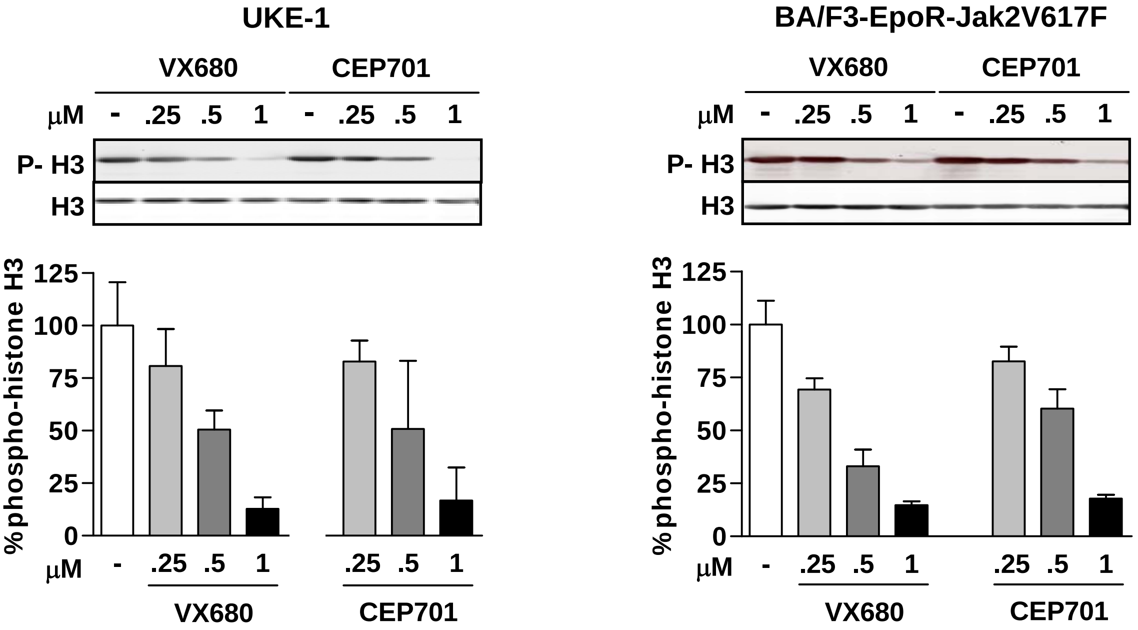

F

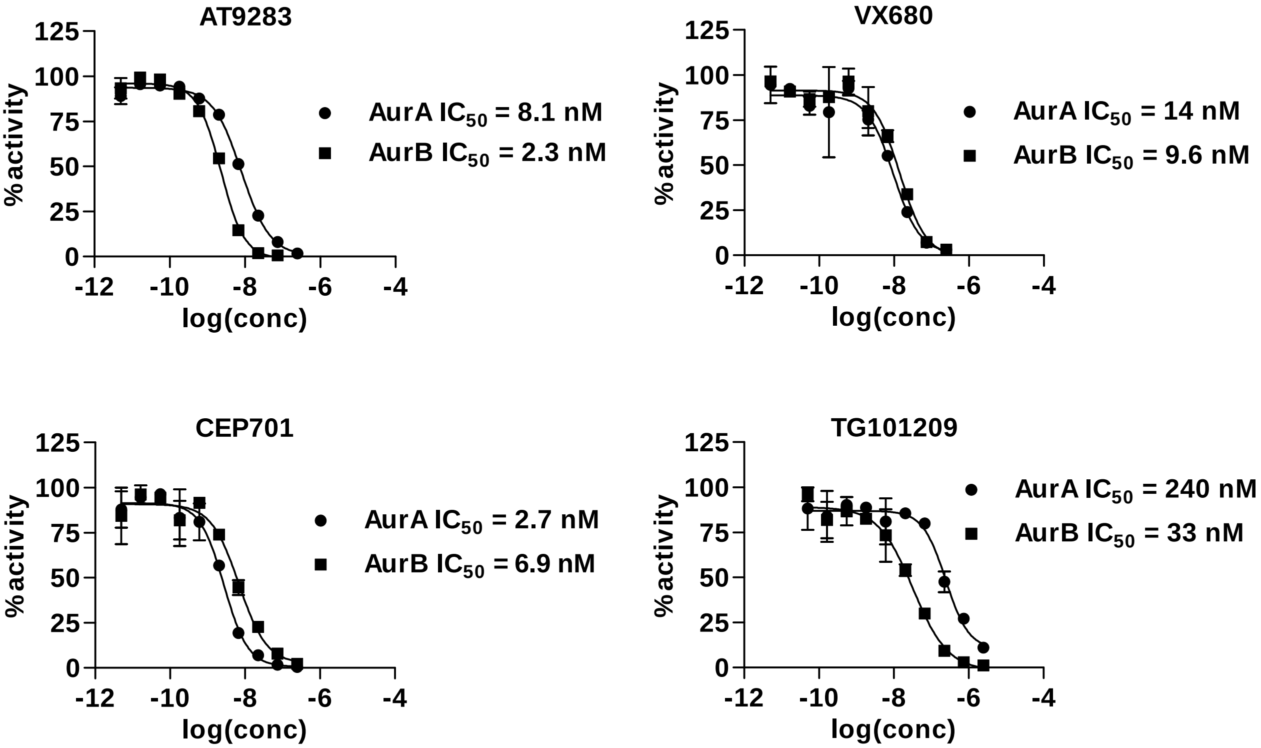

Figure S6E-F: CEP701 has Aurora kinase inhibitory activity.

E, UKE-1 (left panel) and BA/F3-EpoR-Jak2V617F (right panel) cells were treated with 100 ng/ml of Nocodazole for 16 hrs to block the cells in the M phase of the cell cycle. Subsequently, the cells were incubated with different concentrations (0.25, 0.5 or 1 μM) of VX680 and CEP701 or left untreated for 3 hrs. Quantitative Western blot detection was performed as described in Materials and Methods. The blots were detected with antibodies against phosphorylated histone H3 (P-H3) and histone H3 (H3). The bar diagrams below the Western blot representation show the quantitation of phospho-histone H3 signals relative to histone H3 expression for three biological replicates. F, IC_{50} values for Aurora A and B inhibition were determined in an *in vitro* kinase assay for the indicated inhibitors. The assays were performed by Caliper Discovery Alliances & Services "CDAS" applying the LabChip technology from Caliper Life Sciences using standard conditions and Staurosporine as a reference compound. IC_{50} values were determined using GraphPad Prism 5.01, log [inhibitor] vs. response-Variable slope 4PL curve fit from duplicate experiments.

Figure S7

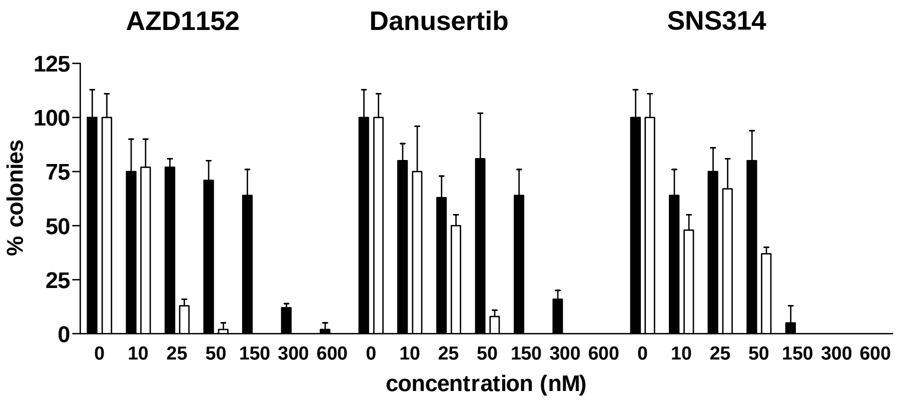

Figure S8

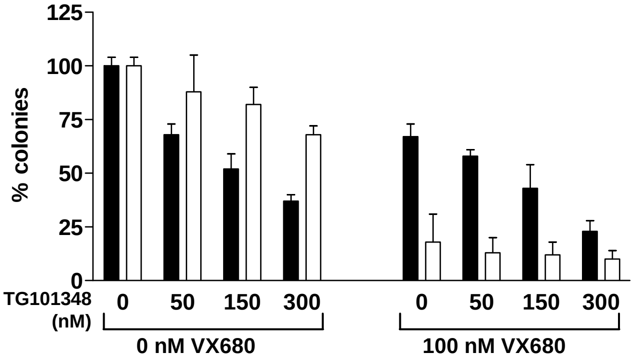

Figure S9

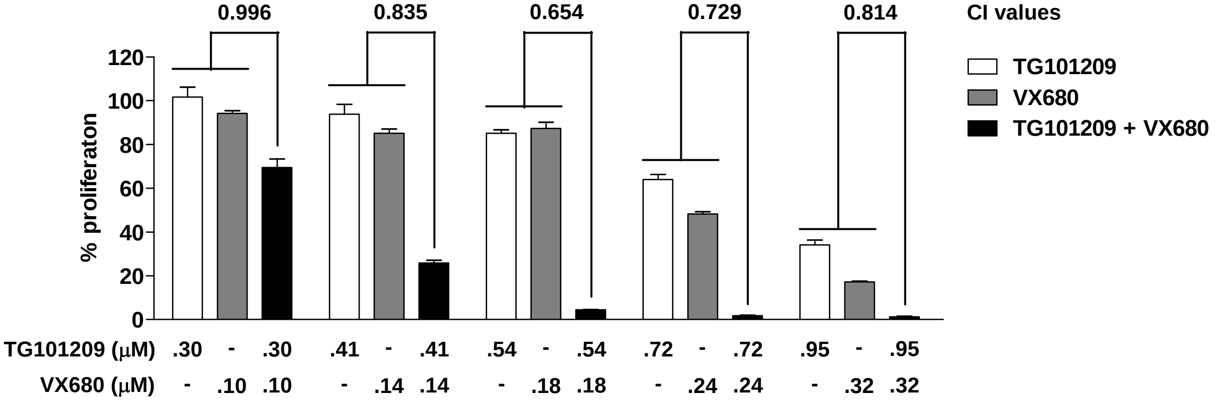

Figure S7: Aurora kinase inhibition rather suppresses growth of myeloid colonies.

$CD34^+$ cells of Jak2V617F-positive MPN patients were subjected to CFC assays in the presence of indicated Aurora kinase inhibitors at different concentrations. The amount of erythroid colonies (black bars) or myeloid colonies (white bars) grown from inhibitor-treated cells was calculated as percentage of maximum number of colonies in the untreated control.

Figure S8: Jak and Aurora kinase inhibitors cooperate to suppress growth of Jak2V617F-expressing cells.

$CD34^+$ cells of Jak2V617F-positive MPN patients were subjected to CFC assays using the Aurora kinase inhibitor VX680 and the Jak2 inhibitor TG101348 in combination. The amount of erythroid colonies (black bars) or myeloid colonies (white bars) grown from inhibitor-treated cells was calculated as percentage of maximum number of colonies in the untreated control.

Figure S9: Chou-Talalay analysis TG101209 and VX680 synergistically inhibit proliferation of BA/F3-EpoR-Jak2V617F cells

BA/F3-EpoR-Jak2V617F cells were incubated with TG101209, VX680 or a combination thereof (ratio of concentrations was kept constant at 3:1). A dose-effect analysis of the drug combination was performed using the CompuSyn software. CI values were lower than 1 for all concentrations indicating a synergistic effect of the drug combination.
